# Supplementary material for: Is liver transplantation ‘out-of-hours’ non-inferior to ‘in-hours’ transplantation? A retrospective analysis of the UK Transplant Registry
Source: BMJ Open. 2019 Feb 20;9(2):e024917. doi: 10.1136/bmjopen-2018-024917 (PMC6398642; doi:10.1136/bmjopen-2018-024917)
Supplement: Supplementary file 1 [file bmjopen-2018-024917supp001.pdf]

Appendix/Supplementary tables

Table A1 Percentage of missing values for each variable of interest, for liver only transplants in the UK, 1st January 2000 to 31st December 2014

**Total number of transplants**	**8816**
	**% missing**
**Recipient characteristics**	
Transplant year	0
Super - urgent	0
Age at transplant, mean (SD)	0
Male gender	0
Caucasian	0
MELD at transplant, mean (SD)	3.2
UKELD at transplant, mean (SD)	3.2
Primary liver disease	0
ABO Blood group	0
Renal support	0.2
In-patient	0.1
Ventilated	0.1
Oesophageal varices	0.4
Presence of TIPS	0
Sepsis confirmed	0.3
Portal vein thrombosis	0
BMI kg/m^2, mean (SD)	3.5
**Donor characteristics**	
Donor age, mean (SD)	0
DCD	0
Split liver	0
Organ appearance suboptimal	0.2
Cause of death	0
**Operative characteristics**	
Night-time procurement	2.3
Night-time transplant	2.5
Weekend transplant	0
CIT (hours), mean (SD)	4.7
Previous abdominal surgery	0.3

Table A2 Risk adjusted hazard ratio from Cox regression model for chance of graft failure or death following liver transplantation in the UK, 1st January 2000 to 31st December 2014

Risk factor	Hazard ratio (95% confidence interval)		
	30 days	1 year	3 years
**Recipient characteristics**			
Transplant year			
2000	1.00 (-)	1.00 (-)	1.00 (-)
2001	1.07 (0.74 - 1.54)	0.94 (0.71 - 1.23)	0.90 (0.71 - 1.14)
2002	0.82 (0.56 - 1.21)	0.76 (0.58 - 1.01)	0.79 (0.62 - 1.01)
2003	0.92 (0.62 - 1.35)	0.90 (0.68 - 1.18)	0.84 (0.66 - 1.07)
2004	0.92 (0.63 - 1.35)	0.96 (0.74 - 1.25)	0.88 (0.70 - 1.11)
2005	0.75 (0.49 - 1.13)	**0.73 (0.54 - 0.98)**	**0.72 (0.56 - 0.93)**
2006	0.75 (0.50 - 1.12)	0.76 (0.58 - 1.02)	**0.75 (0.59 - 0.96)**
2007	0.71 (0.48 - 1.06)	**0.64 (0.48 - 0.86)**	**0.63 (0.49 - 0.81)**
2008	**0.59 (0.38 - 0.89)**	**0.62 (0.46 - 0.82)**	**0.63 (0.49 - 0.80)**
2009	**0.58 (0.38 - 0.88)**	**0.58 (0.43 - 0.78)**	**0.59 (0.46 - 0.76)**
2010	**0.64 (0.42 - 0.96)**	**0.57 (0.42 - 0.76)**	**0.56 (0.43 - 0.72)**
2011	**0.58 (0.38 - 0.88)**	**0.46 (0.33 - 0.62)**	**0.50 (0.38 - 0.65)**
2012	**0.50 (0.32 - 0.76)**	**0.45 (0.33 - 0.62)**	**0.51 (0.39 - 0.66)**
2013	**0.62 (0.42 - 0.92)**	**0.51 (0.39 - 0.69)**	**0.50 (0.38 - 0.65)**
2014	**0.48 (0.31 - 0.73)**	**0.44 (0.33 - 0.60)**	**0.46 (0.34 - 0.60)**
Age at transplant	**1.01 (1.00 - 1.01)**	**1.01 (1.01 - 1.01)**	**1.01 (1.00 - 1.01)**
Caucasian	0.89 (0.71 - 1.11)	0.85 (0.72 - 1.01)	**0.85 (0.74 - 0.97)**
Primary liver disease			
Cancer	1.00 (-)	1.00 (-)	1.00 (-)
HCV	0.79 (0.50 - 1.24)	0.87 (0.65 - 1.17)	0.94 (0.74 - 1.19)
Alcohol related liver disease	1.05 (0.69 - 1.59)	0.89 (0.67 - 1.18)	**0.76 (0.61 - 0.96)**
HBV	1.00 (0.55 - 1.82)	0.96 (0.64 - 1.44)	0.83 (0.59 - 1.17)
PSC	1.17 (0.73 - 1.85)	1.16 (0.85 - 1.59)	1.03 (0.80 - 1.33)
PBC	0.90 (0.57 - 1.44)	0.84 (0.61 - 1.14)	**0.65 (0.50 - 0.84)**
Autoimmune hepatitis	1.06 (0.66 - 1.70)	1.00 (0.73 - 1.38)	0.85 (0.65 - 1.11)
Metabolic liver disease	1.24 (0.75 - 2.05)	1.14 (0.81 - 1.61)	0.91 (0.68 - 1.23)
Acute liver disease	1.56 (0.91 - 2.67)	1.34 (0.91 - 1.96)	1.07 (0.77 - 1.49)
Re-transplants	**1.77 (1.08 - 2.91)**	**1.74 (1.24 - 2.44)**	**1.52 (1.14 - 2.02)**
Other	0.95 (0.55 - 1.65)	1.04 (0.72 - 1.49)	0.83 (0.61 - 1.13)
Renal support	**1.42 (1.13 - 1.80)**	**1.44 (1.21 - 1.71)**	**1.42 (1.22 - 1.67)**
In-patient	1.08 (0.85 - 1.36)	**1.29 (1.10 - 1.50)**	**1.22 (1.07 - 1.40)**
Ventilated	**1.85 (1.36 - 2.51)**	**1.59 (1.26 - 2.01)**	**1.42 (1.14 - 1.76)**
Oesophageal varices	**1.18 (1.00 - 1.39)**	1.09 (0.96 - 1.23)	1.03 (0.93 - 1.14)
Presence of TIPS	1.08 (0.71 - 1.64)	1.20 (0.91 - 1.60)	1.22 (0.96 - 1.56)
Sepsis confirmed	**1.49 (1.14 - 1.95)**	**1.33 (1.08 - 1.63)**	**1.29 (1.07 - 1.56)**
Acute failure grade prior to transplant	1.06 (0.74 - 1.51)	0.81 (0.62 - 1.05)	0.78 (0.61 - 0.99)
**Donor characteristics**			
Donor age (years)	1.00 (0.99 - 1.01)	**1.01 (1.00 - 1.01)**	**1.01 (1.00 - 1.01)**
DCD	**2.21 (1.69 - 2.88)**	**2.00 (1.65 - 2.43)**	**1.84 (1.55 - 2.17)**
Split liver	1.18 (0.73 - 1.93)	**2.12 (1.63 - 2.76)**	**2.01 (1.59 - 2.53)**
Organ appearance suboptimal	**2.32 (1.80 - 2.98)**	**1.64 (1.39 - 1.92)**	**1.48 (1.29 - 1.70)**

Split liver * time (days post-transplant)	**1.05 (1.01 - 1.08)**	0.999 (0.997 - 1.001)	0.999 (0.998 - 1)
Organ appearance suboptimal * time (days post-transplant)	0.98 (0.96 - 1.00)	**0.998 (0.997 - 0.999)**	0.999 (0.999 - 1)
**Operative characteristics**			
CIT (hours)	**1.04 (1.01 - 1.07)**	**1.03 (1.01 - 1.05)**	**1.02 (1.00 - 1.04)**
Previous abdominal surgery	1.24 (0.97 - 1.58)	**1.20 (1.01 - 1.42)**	1.13 (0.97 - 1.31)
Night-time transplant	1.02 (0.85 - 1.23)	1.01 (0.88 - 1.15)	1.00 (0.89 - 1.12)
Weekend transplant	**0.77 (0.66 - 0.91)**	**0.86 (0.77 - 0.97)**	**0.89 (0.81 - 0.99)**

Table A3 Risk adjusted hazard ratio from Cox regression model for chance of graft failure following liver transplantation in the UK, 1st January 2000 to 31st December 2014

**Risk factor**	**Hazard ratio (95% confidence interval)**		
	**30 days**	**1 year**	**3 years**
**Recipient characteristics**			
Transplant year			
2000	1.00 (-)	1.00 (-)	1.00 (-)
2001	1.17 (0.72 - 1.90)	1.01 (0.70 - 1.46)	0.92 (0.67 - 1.26)
2002	0.95 (0.57 - 1.57)	0.80 (0.54 - 1.17)	0.83 (0.60 - 1.14)
2003	1.17 (0.72 - 1.90)	1.05 (0.72 - 1.51)	0.95 (0.69 - 1.30)
2004	0.97 (0.59 - 1.61)	1.10 (0.77 - 1.57)	0.95 (0.70 - 1.29)
2005	0.88 (0.52 - 1.50)	0.93 (0.64 - 1.37)	0.86 (0.62 - 1.19)
2006	1.15 (0.70 - 1.87)	1.12 (0.78 - 1.59)	0.98 (0.72 - 1.34)
2007	1.00 (0.60 - 1.65)	0.86 (0.59 - 1.26)	0.81 (0.59 - 1.12)
2008	0.78 (0.46 - 1.32)	0.82 (0.57 - 1.20)	0.81 (0.59 - 1.10)
2009	0.81 (0.48 - 1.36)	0.86 (0.59 - 1.25)	0.84 (0.61 - 1.14)
2010	0.79 (0.47 - 1.33)	0.70 (0.48 - 1.03)	**0.64 (0.46 - 0.90)**
2011	0.93 (0.56 - 1.53)	**0.62 (0.42 - 0.92)**	**0.59 (0.42 - 0.83)**
2012	0.64 (0.38 - 1.10)	**0.61 (0.41 - 0.90)**	**0.62 (0.45 - 0.86)**
2013	0.91 (0.56 - 1.48)	0.76 (0.53 - 1.10)	**0.71 (0.52 - 0.98)**
2014	0.73 (0.43 - 1.21)	**0.62 (0.42 - 0.91)**	**0.63 (0.45 - 0.89)**
Age at transplant	**1.01 (1.00 - 1.02)**	**1.01 (1.00 - 1.01)**	**1.00 (1.00 - 1.01)**
Caucasian	0.92 (0.70 - 1.21)	0.87 (0.71 - 1.07)	0.87 (0.73 - 1.03)
Primary liver disease			
Cancer	1.00 (-)	1.00 (-)	1.00 (-)
HCV	0.87 (0.51 - 1.48)	1.12 (0.77 - 1.64)	1.30 (0.95 - 1.77)
Alcohol related liver disease	1.13 (0.69 - 1.85)	1.08 (0.75 - 1.56)	0.97 (0.71 - 1.32)
HBV	1.26 (0.64 - 2.49)	1.18 (0.71 - 1.96)	1.11 (0.72 - 1.71)
PSC	1.51 (0.88 - 2.58)	**1.61 (1.09 - 2.38)**	**1.49 (1.07 - 2.07)**
PBC	1.10 (0.64 - 1.89)	1.09 (0.73 - 1.63)	0.90 (0.64 - 1.27)
Autoimmune hepatitis	1.14 (0.65 - 2.01)	1.09 (0.71 - 1.65)	0.95 (0.66 - 1.36)
Metabolic liver disease	1.58 (0.89 - 2.80)	**1.61 (1.06 - 2.46)**	1.34 (0.93 - 1.93)
Acute liver disease	**1.99 (1.04 - 3.83)**	**2.02 (1.23 - 3.31)**	**1.72 (1.12 - 2.65)**
Re-transplants	**2.26 (1.26 - 4.05)**	**2.48 (1.61 - 3.81)**	**2.29 (1.59 - 3.30)**
Other	1.14 (0.61 - 2.15)	1.15 (0.72 - 1.84)	0.93 (0.62 - 1.40)
Renal support	**1.39 (1.03 - 1.86)**	**1.30 (1.03 - 1.63)**	**1.26 (1.03 - 1.54)**
In-patient	0.99 (0.75 - 1.30)	1.11 (0.91 - 1.34)	1.14 (0.96 - 1.35)

Ventilated	**1.59 (1.08 - 2.35)**	**1.41 (1.04 - 1.92)**	1.28 (0.97 - 1.69)
Oesophageal varices	1.15 (0.94 - 1.40)	1.15 (0.99 - 1.33)	1.07 (0.94 - 1.22)
Presence of TIPS	1.39 (0.90 - 2.15)	**1.45 (1.06 - 2.00)**	1.30 (0.97 - 1.74)
Sepsis confirmed	**1.51 (1.08 - 2.10)**	**1.35 (1.03 - 1.75)**	**1.31 (1.04 - 1.67)**
Acute failure grade prior to transplant	0.84 (0.54 - 1.31)	**0.70 (0.50 - 0.98)**	**0.64 (0.47 - 0.86)**
**Donor characteristics**			
Donor age (years)	1 (0.99 - 1)	**1 (1 - 1.01)**	**1.01 (1.00 - 1.01)**
DCD	**2.22 (1.65 - 2.99)**	**2.18 (1.74 - 2.72)**	**2.07 (1.71 - 2.52)**
Split liver	1.13 (0.65 - 1.96)	**2.33 (1.72 - 3.16)**	**2.32 (1.77 - 3.04)**
Organ appearance suboptimal	**2.74 (2.06 - 3.65)**	**1.85 (1.53 - 2.24)**	**1.73 (1.47 - 2.05)**
Split liver * time (days post-transplant)	**1.05 (1.01 - 1.09)**	0.999 (0.996 – 1.001)	0.999 (0.998 – 1)
Organ appearance suboptimal * time (days post-transplant)	**0.96 (0.94 - 0.99)**	0.998 (0.997 – 1)	0.999 (0.999 - 1)
**Operative characteristics**			
CIT (hours)	**1.04 (1.00 - 1.08)**	**1.03 (1.00 - 1.06)**	**1.02 (1.00 - 1.04)**
Previous abdominal surgery	1.26 (0.95 - 1.67)	**1.23 (1.00 - 1.51)**	1.16 (0.97 - 1.40)
Night-time transplant	1.14 (0.91 - 1.41)	1.07 (0.91 - 1.26)	1.11 (0.97 - 1.28)
Weekend transplant	**0.81 (0.67 - 0.97)**	0.87 (0.76 - 1.01)	0.89 (0.79 - 1.01)

Table A4 Cox regression model for the chance of transplant failure following liver transplantation at night-time compared to daytime

**Time from transplant**	**Unadjusted**			**Risk adjusted**		
	**Hazard ratio**	**95% interval**	**p-value**	**Hazard ratio**	**95% interval**	**p-value**
**30 days**	1.254	1.059 - 1.483	0.009	1.021	0.851 - 1.226	0.8
**One year**	1.247	1.104 - 1.408	0.0004	1.007	0.882 - 1.149	0.9
**Three years**	1.187	1.068 - 1.319	0.002	0.995	0.887 - 1.116	0.9

Table A5 Cox regression model for the chance of graft failure following liver transplantation at night-time compared to daytime

**Time from transplant**	**Unadjusted**			**Risk adjusted**		
	**Hazard ratio**	**95% interval**	**p-value**	**Hazard ratio**	**95% interval**	**p-value**
**30 days**	1.279	1.046 - 1.564	0.02	1.137	0.914 - 1.413	0.2
**One year**	1.227	1.058 - 1.424	0.007	1.071	0.912 - 1.259	0.4
**Three years**	1.233	1.082 - 1.406	0.002	1.112	0.965 - 1.282	0.1
