# Supplementary material for: Is liver transplantation ‘out-of-hours’ non-inferior to ‘in-hours’ transplantation? A retrospective analysis of the UK Transplant Registry
Source: BMJ Open. 2019 Feb 20;9(2):e024917. doi: 10.1136/bmjopen-2018-024917 (PMC6398642; doi:10.1136/bmjopen-2018-024917)
Supplement: Author's manuscript [file bmjopen-2018-024917.draft_revisions.pdf]

BMJ Open is committed to open peer review. As part of this commitment we make the peer review history of every article we publish publicly available.

When an article is published we post the peer reviewers' comments and the authors' responses online. We also post the versions of the paper that were used during peer review. These are the versions that the peer review comments apply to.

The versions of the paper that follow are the versions that were submitted during the peer review process. They are not the versions of record or the final published versions. They should not be cited or distributed as the published version of this manuscript.

BMJ Open is an open access journal and the full, final, typeset and author-corrected version of record of the manuscript is available on our site with no access controls, subscription charges or pay-per-view fees (http://bmjopen.bmj.com).

If you have any questions on BMJ Open's open peer review process please email info.bmjopen@bmj.com

BMJ Open

Is liver transplantation 'out-of-hours' non-inferior to 'in-hours' transplantation? A retrospective analysis of the UK transplant registry.

Journal:	*BMJ Open*
Manuscript ID	bmjopen-2018-024917
Article Type:	Research
Date Submitted by the Author:	02-Jul-2018

Complete List of Authors:	Halliday, Neil; University College London, Institute of Liver and Digestive Health; University College London, Institute of Immunity and Transplantation<br>Martin, Kate; NHS Blood and Transplant, Statistics and Clinical Studies<br>Collett, Dave; NHS Blood and Transplant, Statistics and Clinical Audit<br>Allen, Elisa; NHS Blood and Transplant, Statistics and Clinical Studies<br>Thorburn, Douglas; University College London, Institute of Liver and Digestive Health; Royal Free London NHS Trust, Sheila Sherlock Liver Centre

Keywords:	TRANSPLANT MEDICINE, TRANSPLANT SURGERY, Hepatology < INTERNAL MEDICINE, Quality in health care < HEALTH SERVICES ADMINISTRATION & MANAGEMENT

SCHOLARONE™
Manuscripts

For peer review only

Is liver transplantation 'out-of-hours' non-inferior to 'in-hours' transplantation? A retrospective analysis of the UK transplant registry.

Neil Halliday, MBBS[1,3]

Kate Martin, MSc[2]

David Collett, PhD[2]

Elisa Allen, PhD[2]

Douglas Thorburn, MD[1]

1: Sheila Sherlock Liver Centre, Royal Free Hospital and the UCL Institute for Liver and Digestive Health, Pond Street, London

2: Statistics and Clinical Studies, NHS Blood and Transplant, Bristol

3: Institute of Immunity and Transplantation, University College London, London

Corresponding Author: Dr Neil Halliday

neilhalliday@doctors.org.uk

Institute for Liver and Digestive Health

University College London

Upper Third Floor, Royal Free Campus

Rowland Hill Street

London, NW3 2PF

Word count: 3540

For peer review only

Is liver transplantation 'out-of-hours' non-inferior to weekday transplantation? A retrospective analysis of the UK transplant registry.

Objectives: Increased morbidity and mortality have been associated with weekend and night-time clinical activity. We sought to compare the outcomes of liver transplantation between weekdays and weekends and between night time and day time to determine if 'out-of-hours' liver transplantation has acceptable results compared to 'in-hours'.

Design, setting and participants: We conducted a retrospective analysis of patient outcomes from the UK Transplant Registry including all 8816 adult, liver-only transplant recipients in the UK between 2000 and 2014.

Outcome measures: Outcome measures were rates of graft and transplant failure at 30 days, one-, and three-years post transplantation. These were correlated with weekend vs weekday and day vs night transplantation, following the construction of a risk adjusted Cox regression model.

Results: Similar patient and donor characteristics were observed between weekend and weekday transplantation. Unadjusted graft failure estimates were 5.7% at 30 days, 10.4% at one year and 14.6% at three years and transplant failure estimates were 7.9%, 15.3% and 21.3% respectively.

A risk adjusted Cox regression model demonstrated a significantly lower adjusted hazard ratio (95% CI) of transplant failure for weekend transplant of 0.77 (0.66 - 0.91) within 30 days, 0.86 (0.77 - 0.97) within one year, 0.89 (0.81 - 0.99) within three years and for graft failure of 0.81 (0.67 – 0.97) within 30 days. For patients without transplant failure within 30 days, there was no weekend effect on transplant

For peer review only

failure. Neither night-time procurement nor transplantation were associated with an altered hazard of transplant or graft failure.

Conclusions: Weekend and night-time liver transplantation outcomes are non-inferior to weekday or day-time transplantation and we observed a possible beneficial effect of weekend transplantation, dependent upon peri-transplant factors. The structure of liver transplant services in the UK delivers acceptable outcomes 'out-of-hours' and may offer wider lessons for weekend working structures.

Article Summary: Strengths and limitations

- This is the first study to address whether there is a weekend effect upon clinical outcomes for liver transplantation in the UK.
- The study was based on an assessment of a large, unbiased, multicentre dataset of all UK liver transplant recipients occurring in the study period.
- The UK transplant registry is a well curated, highly complete database enabling the generation of risk adjusted models including recipient, donor and technical parameters that may influence outcomes.
- Transplantation settings offer the ability to explore outcomes where the timing of clinical event is not determined by the recipient's clinical status
- The major limitations of this study include the inability to identify causative factors, nor identify confounding factors that may be driving differences in outcomes.

For peer review only

Sources of funding: This research received no specific grant from any funding agency in the public, commercial or not-for-profit sectors. NH is supported by a Wellcome Trust PhD Studentship.

Competing interests: The authors declare no competing interests.

Author contributions: NH designed the study concept and strategy, undertook data analysis and drafted and revised the manuscript. KM proposed statistical methods, undertook data analysis and drafted the manuscript. DC proposed statistical methods, reviewed data analysis and drafted the manuscript. EA proposed statistical methods, undertook data analysis and drafted the manuscript. DT designed the study concept and strategy, undertook data analysis and drafted and revised the manuscript.

Data Sharing: Data and statistical code can be made available on request by enquiry via the corresponding author.

For peer review only

Introduction

Increased morbidity and mortality has been observed with out-of-hours clinical practice in a range of settings[1], which has in part been ascribed to differing clinical service provision through the week. Liver transplantation (LT) services are structured differently to most other clinical services[2] due to the complexity, time sensitivity, scarcity of donations and potential risk of LT. All aspects of LT care are consultant-led, with a standardised service provided at all times and multiple clinical teams including surgeons, anaesthetists, physicians, radiologists and intensive care specialists involved in each case, assisted by specialist co-ordinating staff. Whether this service structure protects against potential weekend effects has not previously been explored in the United Kingdom (UK).

Several studies report excess mortality associated with weekend hospital admission in the UK [3-7] and elsewhere [1,8,9]. However, despite adverse weekend effects being observed in many studies, they are not consistent across all diagnoses and presentations. For example, a Canadian study of nearly 3.8 million hospital admissions [10], a multinational meta-analysis of 251 patient cohorts, comprising over 25 million patients [11], and an Australian study of 3.3 million admissions [12] all reported worse weekend outcomes for some, but not all, diagnoses and presentations. Even within conditions associated with adverse weekend effects, conflicting outcomes have been reported, for example increased mortality has been demonstrated

For peer review only

following weekend admission with stroke or upper gastrointestinal haemorrhage in some [13-15], but not all [16-19] studies. These findings suggest that adverse weekend effects are complex, disease specific and may have different underlying causes including service structure[19]. Despite this, a recent assessment of the impact of enhanced 7 day working practices in the UK did not show a beneficial impact on adverse weekend outcomes [20].

A similar picture of variable weekend effects has been observed with surgery and intensive care admissions. Elective weekend surgical outcomes and emergency surgical presentations are associated with higher mortality in the majority of studies, including large meta-analyses [9,10,21-27] but not in all studies [28,29]. Weekend effects have also been demonstrated in some [30-32] but not all [33] intensive care unit (ICU) admission studies.

It remains unclear whether the observed excess mortality associated with weekend admission is a product of differing case severity between weekdays and weekends [34,35], differing admission thresholds [36], systematic differences in care delivery, structure and staffing of services, quality of care, or poor quality data recording [17] or is an artefact [37]. As weekend effects are specific to different diagnoses and clinical scenarios, if the differences in clinical outcomes are due to service provision and structure each clinical service structure should be tested for acceptable of outcomes across the week.

The current evidence for out-of-hours LT outcomes is mixed. No increased risk of mortality or graft failure was demonstrated with weekend or night-time LT in a multi-

For peer review only

centre American study of nearly 95,000 transplants [38]. Another single centre American study demonstrated no increase in surgical complications or long-term mortality but did show an increase in early mortality following LT at night [39]. Renal transplantation at the weekend was not associated with increased mortality or graft failure in a UK study of nearly 13,000 transplants [40], a smaller German study [41] (although higher rates of surgical complications were observed), or a large American study [42].

The UK delivers LT services with a high volume, low centre-number model, with seven centres providing services for a population of approximately 65 million people, each performing between 30 to 172 deceased donor, adult-recipient LTs annually [43]. With the development of a national organ retrieval service with stipulated retrieval response times and increasing reliance on donation after cardiac death (DCD), the incidence of out-of-hours transplantation has been increasing. We wanted to establish whether the model of service delivery in the UK ensures consistency in outcomes throughout the day and week. We retrospectively assessed the impact of night-time and weekend LT upon recipient death and graft failure following single organ LT across all UK centres.

For peer review only

Methods

Data on all adult recipients (≥17 years) of liver only transplants from deceased donors in the UK under the National Health Service (NHS) between 1st January 2000 and 31st December 2014 were obtained from the UK Transplant Registry and followed up to 18th February 2016.

Night-time procurement was deemed to be any liver donation where the liver perfusion start time was between 7pm and 7am. We estimated transplant operation time by adding donor liver perfusion start time to cold ischaemia time (CIT). Liver perfusion data was not collected before 2000 and so we have only included transplants since 2000 in the cohort. Night-time transplantation was defined as operation time between 7pm and 7am. Weekend transplantation was defined as any transplant operation time between 5pm on a Friday and 8am on a Monday whereas weekday transplantation included all other time points. These time points were selected to ensure that our findings were comparable to other published studies[38].

The primary outcomes were graft failure and transplant failure. Transplant failure was defined as the earlier of graft failure or patient death (graft failure before death, graft failure and death, or death with a functioning graft were classed as an event), whereas graft failure classed graft failure before death or graft failure and death as an event. T-test, chi-square, and log rank tests were used to compare weekday with weekend transplant for continuous, categorical, and failure rate data, respectively. Cox proportional hazards models were built to estimate graft and transplant failure at 30 days, one, and three years post-transplant. Factors considered for inclusion in the model are listed in table 1. Stepwise variable selection, a combination of forwards and backwards selection, was used to identify factors to be included in the models for the different end points.

Less than 5% of values for each baseline patient, donor and operative characteristic were missing (see supplementary table A1). Missing values for the following recipient factors: international normalised ratio (INR), sodium, creatinine, and

For peer review only

bilirubin (used in calculating Model of End-stage Liver Disease (MELD) and United Kingdom model for End-stage Liver Disease (UKELD) score), body mass index (BMI), CIT, on renal support, acute failure grade, in-patient, ventilated, oesophageal varices, sepsis confirmed, previous abdominal surgery; and surgical factors: suboptimal organ appearance, night-time procurement, night-time transplant and weekend transplant were imputed using multiple imputation based on chained equations [44]. This involved generating 11 data sets with imputed values, with the median of continuous variables and the modal value of the categorical variables being used to produce the final data set. These factors were investigated for any pattern of missingness, but there was generally no evidence of systematic difference in missingness for transplant failure, with the exception of donor type. At one year follow up there were more cases of missing patient or graft outcomes for DCD transplants (14%) compared to donor with brainstem death (DBD) transplants (7.7%).

To assess the fit of the models at each endpoint we used the May and Hosmer test [45]. Schoenfeld residuals and the Grambsch and Therneau test were used to test the assumption of proportional hazards. The functional form of each continuous variable in the model was assessed for non-linearity using martingale residual plots from the null model and by fitting spline terms. The predictive ability of the models was summarised using the c-statistic [46].

Patient involvement: Patients, carers and lay people were not directly involved in the design, conduct and analysis of this study, as it is based on routinely collected data from the UK Transplant Registry. However the study was designed to assess

For peer review only

outcomes that are important to patients including graft failure and mortality. The relevance and timeliness of the study was endorsed by the NHS Blood and Transplant Liver Advisory Group, which includes transplant clinicians, patients and lay members

Data supplied to the UK Transplant Registry are validated on receipt to ensure completeness of follow up. Transplant centres are contacted directly if there are validation queries, or to obtain complete data records. Patient survival is confirmed through death registration where possible. All analysis was performed using SAS 9.4 (SAS Institute, Cary NC).

For peer review only

Results

Data were available for 8816 adult LT performed at all UK centres. Follow up information on graft failure or patient death was available at 30 days for all transplants, at one year for 91.4%, and at three years for 76.2% of transplants. Follow up information is obtained from annual follow up appointments with patients, or notification of death.

The mean recipient age (standard deviation (SD)) at transplantation was 50 (13) years, 60% of the population were male and 88% were Caucasian. Alcohol related liver disease was the leading indication for transplantation (20%) followed by chronic viral hepatitis (16%), acute liver failure (12%), re-transplantation (10%), primary biliary cholangitis (10%), primary sclerosing cholangitis (8%), autoimmune hepatitis (8%), primary liver cancer (6%), metabolic liver disease (6%), and other diagnoses

(5%). The donor population were 51% male with a mean average age (SD) of 46 (16) years. The mean CIT (SD) was 9.3 (3) hours. Eleven percent of livers were from DCDs and the remainder from DBDs.

Of the 8816 transplants, 3203 (36.3%) were performed in the weekend period and 6101 (69.2%) at night. Overall unadjusted transplant failure estimates were 7.9% at 30 days, 15.3% at one year, and 21.3% at three years and graft failure estimates were 5.7%, 10.4%, and 14.6%, respectively.

Effect of weekend transplantation

Table 1 summarises the donor and recipient characteristics by weekday and weekend transplantation, which were all tested for inclusion in the model building process. Transplants at the weekend had a higher frequency of factors associated with poorer outcomes including being an in-patient, being listed for super-urgent indications and having active sepsis at the time of transplantation, however more split liver grafts were performed on weekdays. Lower mean average MELD and UKELD scores were seen on weekdays compared to weekends (19.1 vs 19.5 (p=0.05) and 56.0 vs 56.4 (p=0.02) respectively) although the difference is small and may not be clinically meaningful. Night-time procurement and transplantation were more likely at the weekend.

Similar proportions of first- and re-transplant procedures, organs from DCDs or DBDs and livers from paediatric or adult donors were seen between weekend and weekday recipients.

For peer review only

The following factors were found to be non-significant when comparing weekday and weekend transplants, and in the risk-adjusted models: recipient gender, cause of recipient death, cause of graft failure, donor gender, outcome of first offer (decline/accept).

In the unadjusted analysis, graft failure was similar for weekday and weekend transplantation with 6% and 5% graft failure at 30 days respectively, 11% and 10% at one year, and 15% and 14% at three years. Transplant failure was higher amongst weekday recipients at 30 days (8% vs 7% (p=0.01)), but not significantly different at other time points (see Figure 1).

The factors that significantly affected transplant failure at each of the three time points and were included in each model were: recipient factors: on renal support, ventilated, confirmed sepsis at time of transplantation, primary liver disease, age, in-patient at time of transplantation, acute liver failure, previous abdominal surgery, presence of oesophageal varices, presence of TIPS, Caucasian: graft factors: organ appearance suboptimal, CIT, donor age, DCD, split liver and transplant year and night-time transplant. Each model was built separately for the different endpoints and outcomes (graft failure or transplant failure) and any significant factors were included in the risk-adjusted models (Supplementary Tables A2 and A3). The c-statistics for the transplant failure and graft failure models were 0.65, 0.63, and 0.60; and 0.64, 0.62 and 0.60 at 30 days, 1 year, and 3 years, respectively.

The risk adjusted hazard ratio (95% confidence interval) of transplant failure for weekend transplant relative to weekday was 0.77 (0.66 - 0.91) within 30 days. The

For peer review only

corresponding hazard ratios for one year and three years were 0.86 (0.77 - 0.97), 0.89 (0.81 - 0.99), respectively (table 2). A weekend transplant had a significant effect on hazard of graft failure alone at 30 days post-transplant of 0.81 (0.67 – 0.97), and a marginal at one and three years post-transplant (table 3). To ensure that the imputed data did not influence the outcomes, analysis excluding the cases with imputed data (therefore including 8037 cases) revealed a similar pattern of results (data not shown).

Differences in surgical complexity could potentially influence outcomes between time periods, for example if more complex patients were selected for transplantation during the week. There is no direct measure of surgical complexity available, but factors that may reflect this were similar between weekdays and weekends including the mean number of units of blood transfused intra-operatively (5.1 weekdays vs 5.0 weekends (p=0.6)), mean length of in-patient stay (21.9 days and 22.4 days respectively (p=0.24)), presence of portal vein thrombosis (2% vs 2% (p=0.9)), recipient BMI (26.4 vs 26.4kg/m^2 (p=0.5)), presence of TIPS (3% vs 4% (p=0.06)) and prior abdominal surgery (20% vs 21% (p=0.2) although the mean length of ICU stay was longer with weekend transplantation (5.4 (SD 9) vs 6.1 (SD 12) days (p=0.008)).

To explore whether there were specific periods in the week that were associated with increased risk of transplant or graft failure, we tested early and late weekdays and each day individually. There was no observed difference in graft or transplant failure between patients transplanted during the day in the early week (Monday 8am-7pm, Tuesday and Wednesday 7am-7pm) and the late week (Thursday 7am-7pm and

For peer review only

Friday 7am-5pm), and the day of the week did not significantly affect the risk of failure, after risk-adjustment, at any of the three time points. An interaction between weekend and night-time transplant was found to be non-significant (p=0.9 at 30 days, 0.5 at 1 year and 0.7 at 3 years). As there were large differences in the number of transplants performed per centre during the study period (range 533 to 2112) we tested whether there were variations in the frequency of weekend transplantation and in weekend outcomes on a per centre basis. The weekend effect was consistent across all transplant centres, and an interaction between weekend transplant and centre was non-significant (p=0.3 at 30 days, 0.2 at 1 year and 0.1 at 3 years).

We considered whether there were different rates of higher risk organ use between weekends and weekdays. We observed that use of organs previously declined by another centre at their first offering was the same for weekdays (31%) and weekends (32%) (p=0.23) and the proportion declined due to donor factors, as compared to other factors, was identical. Donor age, use of DCDs, CIT and suboptimal organ appearance were similar.

To determine when the factors leading to reduced outcomes following weekday transplantation were operating, analysis was restricted to those who did not suffer death or graft failure prior to 30 days. This resulted in no significant weekend effect on transplant failure after one and three years, indicating that the factors operate in the early peri-transplant period and continue to effect long term failure rates.

Effect of night-time procurement or transplantation

For peer review only

A similar analysis and model building strategy was undertaken for transplants assessed as day-time (operative start time 7am-7pm) compared to night-time and also for day-time compared to night-time organ procurement. 2715 (31%) transplants were undertaken during the day-time and 6101 (69%) at night. Night-time procurement occurred in 6228 (71%) of transplants. Day-time compared to night-time transplant was associated with lower graft failure at 30 days (5% vs 6% (p=0.02)), one year (9% vs 11% (p=0.007)), and three years (13% vs 15% (p=0.002)) and transplant failure at 30 days (7% vs 8% (p=0.008)) and one year (13% vs 16% (p=0.0004)), and three years (19% vs 22% (p=0.001)) in the unadjusted analysis. Day-time compared to night-time procurement was associated with lower graft failure at 30 days (5% vs 6% (p=0.04)), one year (9% vs 11% (p=0.005)), and three years (13% vs 15% (p=0.002)) and transplant failure at 30 days (7% vs 8% (p=0.03)), one year (13% vs 16% (p=0.0007)), and three years (19% vs 22% (p=0.003)) in the unadjusted analysis. In the Cox proportional hazard models, utilising the same variables as for the weekend vs weekday model neither night-time procurement nor night-time transplantation had a significant effect on transplant or graft failure at any time point (Supplementary Tables A4 and A5).

For peer review only

Discussion

We have demonstrated no increased risk of graft or transplant failure with weekend LT, night-time LT or night-time graft procurement. These findings suggest that UK liver transplant outcomes do not have an increased risk of adverse outcomes associated with 'out-of-hours' operating. Interestingly there is a possible reduction in the hazard of early graft failure and long-term transplant failure associated with

weekend transplantation. The loss of this association when considering only survivors at 30 days suggests that any responsible factors are acting in the early peri-transplant period. The effect is present in the unadjusted transplant failure data at 30 days, in the adjusted outcomes at all time points measured, and is seen across all centres.

To explore whether the finding of possible improved weekend outcomes could be explained we tested several hypotheses. Patients transplanted at weekends were not lower risk; in fact they were more likely to have adverse prognostic markers and there were no differences in the causes of liver disease between the two groups. There were no systematic differences in organ utilisation: markers of adverse graft features were similar including average donor ages and the proportion of DCD, suboptimal and previously declined organs that were transplanted. No differences in markers of surgical complexity were noted including portal vein thrombosis, oesophageal varices, re-transplantation, prior abdominal surgery, BMI, and volume of blood transfused intraoperatively. There was no evidence for systemic delaying of operative start times following organ retrieval, as the CIT was similar. If this were occurring to ensure adequate practitioner rest at night or prioritise elective commitments, it would theoretically be seen more in weekday transplantation.

A lead in effect, where patients transplanted in the week are stepped down from the ICU to general wards over the weekend, would disproportionally affect transplants occurring early in the week, however we found no such association. Weekend LT was associated with a longer average ICU stay (6.1 vs 5.4 days) but whether this would improve outcomes, was a marker of a more unwell patient population, or is of

For peer review only

any clinical importance is unclear. Therefore we are unable to explain this association and it may relate to other unmeasured factors in patient or graft selection or care.

We speculate that the senior-clinician led and delivered service delivery may obviate any inherent weekend risks. Although as previously noted, not all clinical scenarios are associated with 'out-of-hours' risks. However this service structure would also not explain difference we observed between weekend and weekday outcomes. It is conceivable that there may be a protective effect of weekend staffing patterns or hospital environment upon LT outcomes, for example, does the absence of routine elective work and competing clinical activity potentially free clinicians and resources for a more prompt and responsive service?

The limitations of this work include that, as with all retrospective registry based studies, neither causality nor aetiological factors can be identified. Furthermore registry data is dependent upon the quality of data imputing, curation, and assignation of variables. However, this is a well curated, highly complete dataset that has previously been used for multiple studies. The definition of day and night in regard to transplantation surgery is artificial as combined organ retrieval, transfer, and implantation straddles both day and night periods but our methodology has been utilised in a previous similar study of LT [38].

This study is an interesting comparator for published disease specific and unselected admissions studies of out-of-hours outcomes [3-17,21-25,28,29]. Our cohort of patients had a standardised risk profile throughout the week unlike unselected admission studies,

For peer review only

as to qualify for LT they had to meet minimum clinical thresholds, assessed by objective validated clinical scores, yet had to be well enough for the procedure to be performed, thus obviating the selection bias inherent in hospital admission studies. Furthermore the availability of an organ rather than a patient's clinical status determined the timing of the admission and clinical intervention, in contrast to unselected admission studies. Finally our study benefited from a detailed, well-curated database of individual patient clinical parameters and outcomes that enabled accurate risk adjustment models to correct for variation in clinical risk on a per patient basis.

The non-inferiority of out-of-hours LT, as seen in other LT and renal transplant studies [38-42], is reassuring and illustrates that the current model of liver transplant provision in the UK provides acceptable outcomes at traditionally perceived periods of clinical risk. However, the potential for improved weekend clinical outcomes differs to other studies on LT [38,39]. A potential beneficial weekend effect is interesting as if working patterns or hospital resources are responsible this represents a model for improved patient care. Direct comparison of surgeons', ICU, and physicians' workload, clinical commitments, and working patterns between weekdays and weekends and comparisons with other, senior-clinician led services will be of interest.

In summary, we have demonstrated that 'out-of-hours' LT outcomes are not worse than for 'in-hours' procedures and potentially weekend liver transplantation may be associated with reduced adverse outcomes. The weekend LT care structure in the UK may represent a model for the design of other critical out-of-hours services.

For peer review only

Furthermore these findings illustrate the complexity of observed weekend effects, which are likely to be dependent on patient selection.

References

1. Hoshijima H, Takeuchi R, Mihara T, et al. Weekend versus weekday admission and short-term mortality: A meta-analysis of 88 cohort studies including 56,934,649 participants. *Medicine.* 2017;96(17): e6685.
2. England N. https://www.england.nhs.uk/publication/liver-transplantation-service-adults/. Accessed 9th April 2018.
3. Freemantle N, Richardson M, Wood J, et al. Weekend hospitalization and additional risk of death: an analysis of inpatient data. *Journal of the Royal Society of Medicine.* 2012;105(2): 74-84.
4. Freemantle N, Ray D, McNulty D, et al. Increased mortality associated with weekend hospital admission: a case for expanded seven day services? *BMJ (Clinical research ed).* 2015;351: h4596.
5. Aylin P, Yunus A, Bottle A, Majeed A, Bell D. Weekend mortality for emergency admissions. A large, multicentre study. *Quality & safety in health care.* 2010;19(3): 213-217.
6. Handel AE, Patel SV, Skingsley A, Bramley K, Sobieski R, Ramagopalan SV. Weekend admissions as an independent predictor of mortality: an analysis of Scottish hospital admissions. *BMJ open.* 2012;2(6).
7. Mohammed MA, Sidhu KS, Rudge G, Stevens AJ. Weekend admission to hospital has a higher risk of death in the elective setting than in the emergency setting: a retrospective database study of national health service hospitals in England. *BMC health services research.* 2012;12: 87.
8. Barba R, Losa JE, Velasco M, Guijarro C, Garcia de Casasola G, Zapatero A. Mortality among adult patients admitted to the hospital on weekends. *European journal of internal medicine.* 2006;17(5): 322-324.
9. Ruiz M, Bottle A, Aylin PP. The Global Comparators project: international comparison of 30-day in-hospital mortality by day of the week. *BMJ quality & safety.* 2015;24(8): 492-504.
10. Bell CM, Redelmeier DA. Mortality among patients admitted to hospitals on weekends as compared with weekdays. *The New England journal of medicine.* 2001;345(9): 663-668.
11. Zhou Y, Li W, Herath C, et al. Off-Hour Admission and Mortality Risk for 28 Specific Diseases: A Systematic Review and Meta-Analysis of 251 Cohorts. *Journal of the American Heart Association.* 2016;5(3): e003102.
12. Concha OP, Gallego B, Hillman K, Delaney GP, Coiera E. Do variations in hospital mortality patterns after weekend admission reflect reduced quality of care or different patient cohorts? A population-based study. *BMJ quality & safety.* 2014;23(3): 215-222.
13. Ananthakrishnan AN, McGinley EL, Saeian K. Outcomes of weekend admissions for upper gastrointestinal hemorrhage: a nationwide analysis. *Clinical gastroenterology and hepatology : the official clinical practice journal of the American Gastroenterological Association.* 2009;7(3): 296-302e291.

For peer review only

14. Shaheen AA, Kaplan GG, Myers RP. Weekend versus weekday admission and mortality from gastrointestinal hemorrhage caused by peptic ulcer disease. *Clinical gastroenterology and hepatology : the official clinical practice journal of the American Gastroenterological Association.* 2009;7(3): 303-310.
15. Roberts SE, Thorne K, Akbari A, Samuel DG, Williams JG. Mortality following Stroke, the Weekend Effect and Related Factors: Record Linkage Study. *PLoS One.* 2015;10(6): e0131836.
16. Jairath V, Kahan BC, Logan RF, et al. Mortality from acute upper gastrointestinal bleeding in the United kingdom: does it display a "weekend effect"? *Am J Gastroenterol.* 2011;106(9): 1621-1628.
17. Li L, Rothwell PM. Biases in detection of apparent "weekend effect" on outcome with administrative coding data: population based study of stroke. *BMJ (Clinical research ed).* 2016;353: i2648.
18. Murray IA, Dalton HR, Stanley AJ, et al. International prospective observational study of upper gastrointestinal haemorrhage: Does weekend admission affect outcome? *United European gastroenterology journal.* 2017;5(8): 1082-1089.
19. Gupta A, Agarwal R, Ananthakrishnan AN. "Weekend Effect" in Patients With Upper Gastrointestinal Hemorrhage: A Systematic Review and Meta-analysis. *Am J Gastroenterol.* 2017.
20. Meacock R, Sutton M. Elevated mortality among weekend hospital admissions is not associated with adoption of seven day clinical standards. *Emergency medicine journal : EMJ.* 2017.
21. Aylin P, Alexandrescu R, Jen MH, Mayer EK, Bottle A. Day of week of procedure and 30 day mortality for elective surgery: retrospective analysis of hospital episode statistics. *BMJ (Clinical research ed).* 2013;346: f2424.
22. McIsaac DI, Bryson GL, van Walraven C. Elective, major noncardiac surgery on the weekend: a population-based cohort study of 30-day mortality. *Medical care.* 2014;52(6): 557-564.
23. Zapf MA, Kothari AN, Markossian T, et al. The "weekend effect" in urgent general operative procedures. *Surgery.* 2015;158(2): 508-514.
24. Groves EM, Khoshchehreh M, Le C, Malik S. Effects of weekend admission on the outcomes and management of ruptured aortic aneurysms. *Journal of vascular surgery.* 2014;60(2): 318-324.
25. Goldstein SD, Papandria DJ, Aboagye J, et al. The "weekend effect" in pediatric surgery - increased mortality for children undergoing urgent surgery during the weekend. *Journal of pediatric surgery.* 2014;49(7): 1087-1091.
26. Metcalfe D, Castillo-Angeles M, Rios-Diaz AJ, Havens JM, Haider A, Salim A. Is there a "weekend effect" in emergency general surgery? *The Journal of surgical research.* 2018;222: 219-224.
27. Smith SA, Yamamoto JM, Roberts DJ, et al. Weekend Surgical Care and Postoperative Mortality: A Systematic Review and Meta-Analysis of Cohort Studies. *Medical care.* 2017.
28. Knudsen NV, Moller MH. Association of mortality with out-of-hours admission in patients with perforated peptic ulcer. *Acta anaesthesiologica Scandinavica.* 2015;59(2): 248-254.
29. McVay DP, Walker AS, Nelson DW, Porta CR, Causey MW, Brown TA. The weekend effect: does time of admission impact management and outcomes of small bowel obstruction? *Gastroenterology report.* 2014;2(3): 221-225.

For peer review only

30. Barnett MJ, Kaboli PJ, Sirio CA, Rosenthal GE. Day of the week of intensive care admission and patient outcomes: a multisite regional evaluation. *Medical care.* 2002;40(6): 530-539.
31. Cavallazzi R, Marik PE, Hirani A, Pachinburavan M, Vasu TS, Leiby BE. Association between time of admission to the ICU and mortality: a systematic review and metaanalysis. *Chest.* 2010;138(1): 68-75.
32. Galloway M, Hegarty A, McGill S, Arulkumaran N, Brett SJ, Harrison D. The Effect of ICU Out-of-Hours Admission on Mortality: A Systematic Review and Meta-Analysis. *Critical care medicine.* 2017.
33. Arabi Y, Alshimemeri A, Taher S. Weekend and weeknight admissions have the same outcome of weekday admissions to an intensive care unit with onsite intensivist coverage. *Critical care medicine.* 2006;34(3): 605-611.
34. Anselmi L, Meacock R, Kristensen SR, Doran T, Sutton M. Arrival by ambulance explains variation in mortality by time of admission: retrospective study of admissions to hospital following emergency department attendance in England. *BMJ quality & safety.* 2017;26(8): 613-621.
35. Walker AS, Mason A, Quan TP, et al. Mortality risks associated with emergency admissions during weekends and public holidays: an analysis of electronic health records. *Lancet (London, England).* 2017;390(10089): 62-72.
36. Meacock R, Anselmi L, Kristensen SR, Doran T, Sutton M. Higher mortality rates amongst emergency patients admitted to hospital at weekends reflect a lower probability of admission. *Journal of health services research & policy.* 2017;22(1): 12-19.
37. Bray BD, Steventon A. What have we learnt after 15 years of research into the 'weekend effect'? *BMJ quality & safety.* 2017;26(8): 607-610.
38. Orman ES, Hayashi PH, Dellon ES, Gerber DA, Barritt ASt. Impact of nighttime and weekend liver transplants on graft and patient outcomes. *Liver Transpl.* 2012;18(5): 558-565.
39. Lonze BE, Parsikia A, Feyssa EL, et al. Operative start times and complications after liver transplantation. *Am J Transplant.* 2010;10(8): 1842-1849.
40. Anderson BM, Mytton JL, Evison F, Ferro CJ, Sharif A. Outcomes After Weekend Admission for Deceased Donor Kidney Transplantation: A Population Cohort Study. *Transplantation.* 2017;101(9): 2244-2252.
41. Schutte-Nutgen K, Tholking G, Dahmen M, et al. Is there a "weekend effect" in kidney transplantation? *PLoS One.* 2017;12(12): e0190227.
42. Baid-Agrawal S, Martus P, Feldman H, Kramer H. Weekend versus weekday transplant surgery and outcomes after kidney transplantation in the USA: a retrospective national database analysis. *BMJ open.* 2016;6(4): e010482.
43. Martin K, Allen E, Johnson R, Transplant NBa. Annual report on liver transplantation: report for 2014/2015. 2015. http://www.odt.nhs.uk/pdf/organ_specific_report_liver_2015.pdf Accessed June 2016.
44. White IR, Royston P, Wood AM. Multiple imputation using chained equations: Issues and guidance for practice. *Statistics in medicine.* 2011;30(4): 377-399.
45. May S, Hosmer DW. A simplified method of calculating an overall goodness-of-fit test for the Cox proportional hazards model. *Lifetime data analysis.* 1998;4(2): 109-120.
46. Collett D. *Modelling survival data in medical research.* London: Chapman & Hall; 2015.

For peer review only

Tables

Table 1 Characteristics of weekday and weekend liver only transplants in the UK, 1st January 2000 to 31st December 2014

	Weekday transplant	Weekend transplant	p-value
	N (%) unless otherwise stated		
**Total number of transplants**	**5613 (64)**	**3203 (36)**	
**Recipient characteristics**			
Transplant year			
2000	312 (6)	209 (7)	
2001	345 (6)	188 (6)	
2002	335 (6)	232 (7)	
2003	288 (5)	207 (6)	
2004	340 (6)	239 (7)	
2005	315 (6)	172 (5)	
2006	321 (6)	196 (6)	0.002
2007	341 (6)	198 (6)	
2008	379 (7)	195 (6)	
2009	379 (7)	187 (6)	
2010	379 (7)	212 (7)	
2011	406 (7)	209 (7)	
2012	441 (8)	227 (7)	
2013	511 (9)	246 (8)	
2014	521 (9)	286 (9)	
Super - urgent	803 (14)	523 (16)	0.01
Age at transplant, mean (SD)	50 (13)	50.2 (12)	0.5
Male gender	3405 (61)	1899 (59)	0.2
Caucasian	4922 (88)	2814 (88)	0.8
MELD at transplant, mean (SD)	19.1 (9)	19.5 (9)	0.05
UKELD at transplant, mean (SD)	56.0 (6)	56.4 (7)	0.02
Primary liver disease			
Cancer	320 (6)	171 (5)	
HCV	746 (13)	395 (12)	
Alcohol related liver disease	1180 (21)	626 (20)	
HBV	153 (3)	107 (3)	
PSC	451 (8)	276 (9)	0.14
PBC	520 (9)	333 (10)	
Autoimmune hepatitis	453 (8)	255 (8)	
Metabolic liver disease	316 (6)	172 (5)	
Acute liver disease	647 (12)	414 (13)	
Re-transplants	539 (10)	304 (9)	
Other	288 (5)	150 (5)	
ABO Blood group			
O	2405 (43)	1318 (41)	
A	2338 (42)	1341 (42)	0.01
B	648 (12)	372 (12)	
AB	222 (4)	172 (5)	
Renal support	690 (12)	421 (13)	0.2
In-patient	1585 (28)	975 (30)	0.03
Ventilated	585 (10)	373 (12)	0.08

For peer review only

Oesophageal varices	3406 (61)	1991 (62)	0.17
Presence of TIPS	185 (3)	130 (4)	0.06
Sepsis confirmed	224 (4)	175 (5)	0.001
Portal vein thrombosis	104 (2)	61 (2)	0.9
BMI kg/m^2, mean (SD)	26.4 (5)	26.4 (5)	0.5
**Donor characteristics**			
Donor age, mean (SD)	46.4 (15)	46 (16)	0.3
DCD	596 (11)	333 (10)	0.7
Split liver	398 (7)	190 (6)	0.04
Organ appearance suboptimal	1208 (22)	653 (20)	0.2
Cause of death			
Cerebrovascular Accident (CVA)	3736 (67)	2174 (68)	
Miscellaneous	1186 (21)	572 (18)	0.0002
Road Traffic Accident (RTA)	410 (7)	293 (9)	
Other trauma	281 (5)	164 (5)	
**Operative characteristics**			
Night-time procurement	3799 (68)	2429 (76)	<0.0001
Night-time transplant	3715 (66)	2386 (74)	<0.0001
CIT (hours), mean (SD)	9.3 (3)	9.4 (3)	0.24
Previous abdominal surgery	1115 (20)	670 (21)	0.2
**Failure**			
Overall graft failure (%)			
30 day	6	5	0.08
One year	11	10	0.16
Three years	15	14	0.16
Overall transplant failure (%)			
30 day	8	7	0.01
One year	16	14	0.09
Three years	22	20	0.12
**Cause of death at 30 days**	**229 (4)**	**120 (4)**	
Cardiothoracic / Myocardial ischaemia and infarction	29 (13)	17 (14)	
Cerebrovascular accident	11 (5)	2 (2)	
Haemorrhage	15 (7)	7 (6)	0.3
Infection / Septicaemia	32 (14)	15 (13)	
Multi-system failure	67 (29)	47 (39)	
Other	75 (33)	32 (27)	
**Cause of graft failure at 30 days**	**314 (6)**	**152 (5)**	
Biliary complications	6 (2)	4 (3)	
Hepatic artery thrombosis	95 (30)	42 (28)	
Non-thrombotic infarction	14 (4)	12 (8)	0.6
Primary non-function	93 (30)	50 (33)	
Rejection	7 (2)	3 (2)	
Other	99 (32)	41 (27)	

For peer review only

Table 2 **Cox regression model for chance of transplant failure following liver transplantation at the weekend compared to a weekday.**

Time from transplant	Unadjusted			Risk adjusted		
	Hazard ratio	95% interval	p-value	Hazard ratio	95% interval	p-value
**30 days**	0.818	0.698 - 0.959	0.01	0.772	0.658 - 0.906	0.001
**One year**	0.906	0.809 – 1.014	0.09	0.864	0.771 - 0.968	0.01
**Three years**	0.925	0.839 – 1.020	0.12	0.893	0.809 - 0.986	0.02

For peer review only

Table 3 **Cox regression model for chance of graft failure following liver transplantation at the weekend compared to a weekday.**

Time from transplant	Unadjusted			Risk adjusted		
	Hazard ratio	95% interval	p-value	Hazard ratio	95% interval	p-value
**30 days**	0.845	0.700 – 1.020	0.08	0.805	0.665 – 0.973	0.02
**One year**	0.905	0.787 – 1.039	0.16	0.874	0.760 – 1.005	0.06
**Three years**	0.918	0.814 – 1.036	0.16	0.892	0.790 – 1.007	0.06

For peer review only

Figure Legend

Figure 1 Unadjusted transplant failure up to three years post-transplant

For peer review only

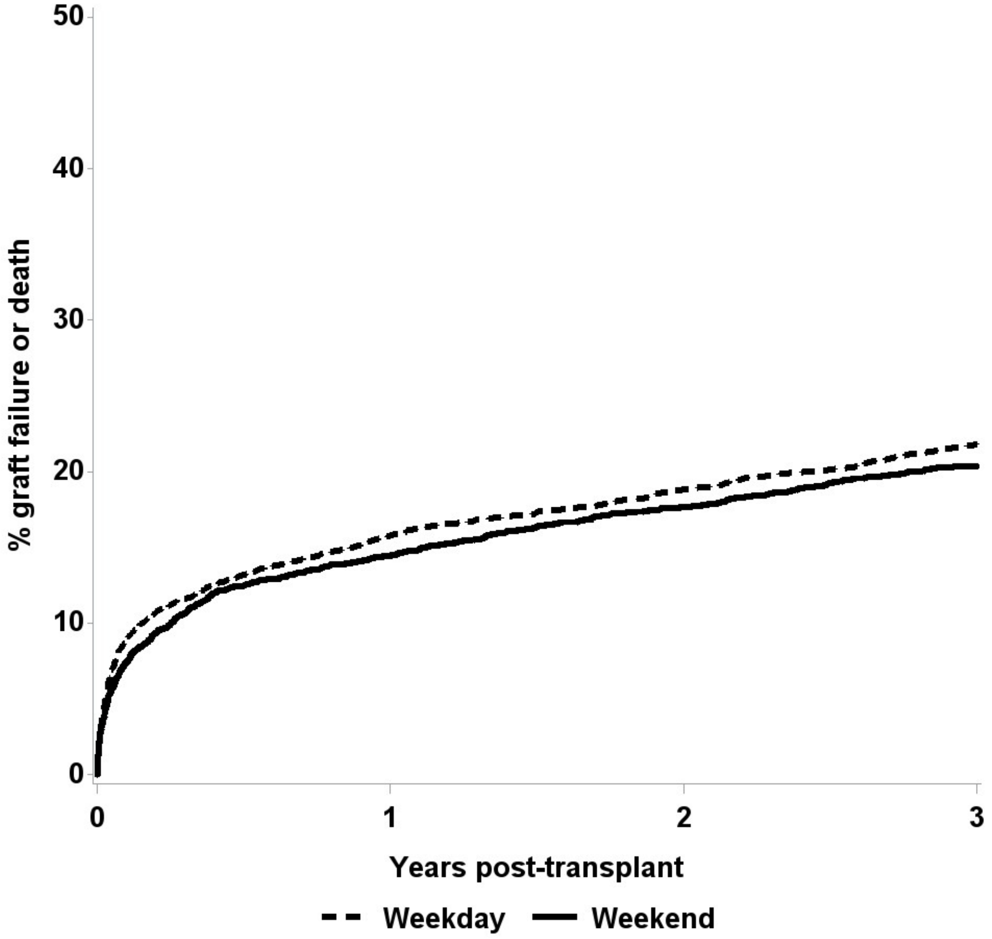

Figure 1 Unadjusted transplant failure up to three years post-transplant

81x81mm (300 x 300 DPI)

Appendix/Supplementary tables

Table A1 Percentage of missing values for each variable of interest, for liver only transplants in the UK, 1st January 2000 to 31st December 2014

**Total number of transplants**	**8816**
	**% missing**
**Recipient characteristics**	
Transplant year	0
Super - urgent	0
Age at transplant, mean (SD)	0
Male gender	0
Caucasian	0
MELD at transplant, mean (SD)	3.2
UKELD at transplant, mean (SD)	3.2
Primary liver disease	0
ABO Blood group	0
Renal support	0.2
In-patient	0.1
Ventilated	0.1
Oesophageal varices	0.4
Presence of TIPS	0
Sepsis confirmed	0.3
Portal vein thrombosis	0
BMI kg/m^2, mean (SD)	3.5
**Donor characteristics**	
Donor age, mean (SD)	0
DCD	0
Split liver	0
Organ appearance suboptimal	0.2
Cause of death	0
**Operative characteristics**	
Night-time procurement	2.3
Night-time transplant	2.5
Weekend transplant	0
CIT (hours), mean (SD)	4.7
Previous abdominal surgery	0.3

For peer review only

Table A2 Risk adjusted hazard ratio from Cox regression model for chance of graft failure or death following liver transplantation in the UK, 1st January 2000 to 31st December 2014

Risk factor	Hazard ratio (95% confidence interval) 30 days	1 year	3 years
**Recipient characteristics**			
Transplant year			
2000	1.00 (-)	1.00 (-)	1.00 (-)
2001	1.07 (0.74 - 1.54)	0.94 (0.71 - 1.23)	0.90 (0.71 - 1.14)
2002	0.82 (0.56 - 1.21)	0.76 (0.58 - 1.01)	0.79 (0.62 - 1.01)
2003	0.92 (0.62 - 1.35)	0.90 (0.68 - 1.18)	0.84 (0.66 - 1.07)
2004	0.92 (0.63 - 1.35)	0.96 (0.74 - 1.25)	0.88 (0.70 - 1.11)
2005	0.75 (0.49 - 1.13)	**0.73 (0.54 - 0.98)**	**0.72 (0.56 - 0.93)**
2006	0.75 (0.50 - 1.12)	0.76 (0.58 - 1.02)	**0.75 (0.59 - 0.96)**
2007	0.71 (0.48 - 1.06)	**0.64 (0.48 - 0.86)**	**0.63 (0.49 - 0.81)**
2008	**0.59 (0.38 - 0.89)**	**0.62 (0.46 - 0.82)**	**0.63 (0.49 - 0.80)**
2009	**0.58 (0.38 - 0.88)**	**0.58 (0.43 - 0.78)**	**0.59 (0.46 - 0.76)**
2010	**0.64 (0.42 - 0.96)**	**0.57 (0.42 - 0.76)**	**0.56 (0.43 - 0.72)**
2011	**0.58 (0.38 - 0.88)**	**0.46 (0.33 - 0.62)**	**0.50 (0.38 - 0.65)**
2012	**0.50 (0.32 - 0.76)**	**0.45 (0.33 - 0.62)**	**0.51 (0.39 - 0.66)**
2013	**0.62 (0.42 - 0.92)**	**0.51 (0.39 - 0.69)**	**0.50 (0.38 - 0.65)**
2014	**0.48 (0.31 - 0.73)**	**0.44 (0.33 - 0.60)**	**0.46 (0.34 - 0.60)**
Age at transplant	**1.01 (1.00 - 1.01)**	**1.01 (1.01 - 1.01)**	**1.01 (1.00 - 1.01)**
Caucasian	0.89 (0.71 - 1.11)	0.85 (0.72 - 1.01)	**0.85 (0.74 - 0.97)**
Primary liver disease			
Cancer	1.00 (-)	1.00 (-)	1.00 (-)
HCV	0.79 (0.50 - 1.24)	0.87 (0.65 - 1.17)	0.94 (0.74 - 1.19)
Alcohol related liver disease	1.05 (0.69 - 1.59)	0.89 (0.67 - 1.18)	**0.76 (0.61 - 0.96)**
HBV	1.00 (0.55 - 1.82)	0.96 (0.64 - 1.44)	0.83 (0.59 - 1.17)
PSC	1.17 (0.73 - 1.85)	1.16 (0.85 - 1.59)	1.03 (0.80 - 1.33)
PBC	0.90 (0.57 - 1.44)	0.84 (0.61 - 1.14)	**0.65 (0.50 - 0.84)**
Autoimmune hepatitis	1.06 (0.66 - 1.70)	1.00 (0.73 - 1.38)	0.85 (0.65 - 1.11)
Metabolic liver disease	1.24 (0.75 - 2.05)	1.14 (0.81 - 1.61)	0.91 (0.68 - 1.23)
Acute liver disease	1.56 (0.91 - 2.67)	1.34 (0.91 - 1.96)	1.07 (0.77 - 1.49)
Re-transplants	**1.77 (1.08 - 2.91)**	**1.74 (1.24 - 2.44)**	**1.52 (1.14 - 2.02)**
Other	0.95 (0.55 - 1.65)	1.04 (0.72 - 1.49)	0.83 (0.61 - 1.13)
Renal support	**1.42 (1.13 - 1.80)**	**1.44 (1.21 - 1.71)**	**1.42 (1.22 - 1.67)**
In-patient	1.08 (0.85 - 1.36)	**1.29 (1.10 - 1.50)**	**1.22 (1.07 - 1.40)**
Ventilated	**1.85 (1.36 - 2.51)**	**1.59 (1.26 - 2.01)**	**1.42 (1.14 - 1.76)**
Oesophageal varices	**1.18 (1.00 - 1.39)**	1.09 (0.96 - 1.23)	1.03 (0.93 - 1.14)
Presence of TIPS	1.08 (0.71 - 1.64)	1.20 (0.91 - 1.60)	1.22 (0.96 - 1.56)
Sepsis confirmed	**1.49 (1.14 - 1.95)**	**1.33 (1.08 - 1.63)**	**1.29 (1.07 - 1.56)**
Acute failure grade prior to transplant	1.06 (0.74 - 1.51)	0.81 (0.62 - 1.05)	0.78 (0.61 - 0.99)
**Donor characteristics**			
Donor age (years)	1.00 (0.99 - 1.01)	**1.01 (1.00 - 1.01)**	**1.01 (1.00 - 1.01)**
DCD	**2.21 (1.69 - 2.88)**	**2.00 (1.65 - 2.43)**	**1.84 (1.55 - 2.17)**
Split liver	1.18 (0.73 - 1.93)	**2.12 (1.63 - 2.76)**	**2.01 (1.59 - 2.53)**
Organ appearance suboptimal	**2.32 (1.80 - 2.98)**	**1.64 (1.39 - 1.92)**	**1.48 (1.29 - 1.70)**

For peer review only

Split liver * time (days post-transplant)	**1.05 (1.01 - 1.08)**	0.999 (0.997 - 1.001)	0.999 (0.998 - 1)
Organ appearance suboptimal * time (days post-transplant)	0.98 (0.96 - 1.00)	**0.998 (0.997 - 0.999)**	0.999 (0.999 - 1)
**Operative characteristics**			
CIT (hours)	**1.04 (1.01 - 1.07)**	**1.03 (1.01 - 1.05)**	**1.02 (1.00 - 1.04)**
Previous abdominal surgery	1.24 (0.97 - 1.58)	**1.20 (1.01 - 1.42)**	1.13 (0.97 - 1.31)
Night-time transplant	1.02 (0.85 - 1.23)	1.01 (0.88 - 1.15)	1.00 (0.89 - 1.12)
Weekend transplant	**0.77 (0.66 - 0.91)**	**0.86 (0.77 - 0.97)**	**0.89 (0.81 - 0.99)**

Table A3 Risk adjusted hazard ratio from Cox regression model for chance of graft failure following liver transplantation in the UK, 1st January 2000 to 31st December 2014

**Risk factor**	**Hazard ratio (95% confidence interval)**		
	**30 days**	**1 year**	**3 years**
**Recipient characteristics**			
Transplant year			
2000	1.00 (-)	1.00 (-)	1.00 (-)
2001	1.17 (0.72 - 1.90)	1.01 (0.70 - 1.46)	0.92 (0.67 - 1.26)
2002	0.95 (0.57 - 1.57)	0.80 (0.54 - 1.17)	0.83 (0.60 - 1.14)
2003	1.17 (0.72 - 1.90)	1.05 (0.72 - 1.51)	0.95 (0.69 - 1.30)
2004	0.97 (0.59 - 1.61)	1.10 (0.77 - 1.57)	0.95 (0.70 - 1.29)
2005	0.88 (0.52 - 1.50)	0.93 (0.64 - 1.37)	0.86 (0.62 - 1.19)
2006	1.15 (0.70 - 1.87)	1.12 (0.78 - 1.59)	0.98 (0.72 - 1.34)
2007	1.00 (0.60 - 1.65)	0.86 (0.59 - 1.26)	0.81 (0.59 - 1.12)
2008	0.78 (0.46 - 1.32)	0.82 (0.57 - 1.20)	0.81 (0.59 - 1.10)
2009	0.81 (0.48 - 1.36)	0.86 (0.59 - 1.25)	0.84 (0.61 - 1.14)
2010	0.79 (0.47 - 1.33)	0.70 (0.48 - 1.03)	**0.64 (0.46 - 0.90)**
2011	0.93 (0.56 - 1.53)	**0.62 (0.42 - 0.92)**	**0.59 (0.42 - 0.83)**
2012	0.64 (0.38 - 1.10)	**0.61 (0.41 - 0.90)**	**0.62 (0.45 - 0.86)**
2013	0.91 (0.56 - 1.48)	0.76 (0.53 - 1.10)	**0.71 (0.52 - 0.98)**
2014	0.73 (0.43 - 1.21)	**0.62 (0.42 - 0.91)**	**0.63 (0.45 - 0.89)**
Age at transplant	**1.01 (1.00 - 1.02)**	**1.01 (1.00 - 1.01)**	**1.00 (1.00 - 1.01)**
Caucasian	0.92 (0.70 - 1.21)	0.87 (0.71 - 1.07)	0.87 (0.73 - 1.03)
Primary liver disease			
Cancer	1.00 (-)	1.00 (-)	1.00 (-)
HCV	0.87 (0.51 - 1.48)	1.12 (0.77 - 1.64)	1.30 (0.95 - 1.77)
Alcohol related liver disease	1.13 (0.69 - 1.85)	1.08 (0.75 - 1.56)	0.97 (0.71 - 1.32)
HBV	1.26 (0.64 - 2.49)	1.18 (0.71 - 1.96)	1.11 (0.72 - 1.71)
PSC	1.51 (0.88 - 2.58)	**1.61 (1.09 - 2.38)**	**1.49 (1.07 - 2.07)**
PBC	1.10 (0.64 - 1.89)	1.09 (0.73 - 1.63)	0.90 (0.64 - 1.27)
Autoimmune hepatitis	1.14 (0.65 - 2.01)	1.09 (0.71 - 1.65)	0.95 (0.66 - 1.36)
Metabolic liver disease	1.58 (0.89 - 2.80)	**1.61 (1.06 - 2.46)**	1.34 (0.93 - 1.93)
Acute liver disease	**1.99 (1.04 - 3.83)**	**2.02 (1.23 - 3.31)**	**1.72 (1.12 - 2.65)**
Re-transplants	**2.26 (1.26 - 4.05)**	**2.48 (1.61 - 3.81)**	**2.29 (1.59 - 3.30)**
Other	1.14 (0.61 - 2.15)	1.15 (0.72 - 1.84)	0.93 (0.62 - 1.40)
Renal support	**1.39 (1.03 - 1.86)**	**1.30 (1.03 - 1.63)**	**1.26 (1.03 - 1.54)**
In-patient	0.99 (0.75 - 1.30)	1.11 (0.91 - 1.34)	1.14 (0.96 - 1.35)

For peer review only

Ventilated	**1.59 (1.08 - 2.35)**	**1.41 (1.04 - 1.92)**	1.28 (0.97 - 1.69)
Oesophageal varices	1.15 (0.94 - 1.40)	1.15 (0.99 - 1.33)	1.07 (0.94 - 1.22)
Presence of TIPS	1.39 (0.90 - 2.15)	**1.45 (1.06 - 2.00)**	1.30 (0.97 - 1.74)
Sepsis confirmed	**1.51 (1.08 - 2.10)**	**1.35 (1.03 - 1.75)**	**1.31 (1.04 - 1.67)**
Acute failure grade prior to transplant	0.84 (0.54 - 1.31)	**0.70 (0.50 - 0.98)**	**0.64 (0.47 - 0.86)**
**Donor characteristics**			
Donor age (years)	1 (0.99 - 1)	**1 (1 - 1.01)**	**1.01 (1.00 - 1.01)**
DCD	**2.22 (1.65 - 2.99)**	**2.18 (1.74 - 2.72)**	**2.07 (1.71 - 2.52)**
Split liver	1.13 (0.65 - 1.96)	**2.33 (1.72 - 3.16)**	**2.32 (1.77 - 3.04)**
Organ appearance suboptimal	**2.74 (2.06 - 3.65)**	**1.85 (1.53 - 2.24)**	**1.73 (1.47 - 2.05)**
Split liver * time (days post-transplant)	**1.05 (1.01 - 1.09)**	0.999 (0.996 – 1.001)	0.999 (0.998 – 1)
Organ appearance suboptimal * time (days post-transplant)	**0.96 (0.94 - 0.99)**	0.998 (0.997 – 1)	0.999 (0.999 - 1)
**Operative characteristics**			
CIT (hours)	**1.04 (1.00 - 1.08)**	**1.03 (1.00 - 1.06)**	**1.02 (1.00 - 1.04)**
Previous abdominal surgery	1.26 (0.95 - 1.67)	**1.23 (1.00 - 1.51)**	1.16 (0.97 - 1.40)
Night-time transplant	1.14 (0.91 - 1.41)	1.07 (0.91 - 1.26)	1.11 (0.97 - 1.28)
Weekend transplant	**0.81 (0.67 - 0.97)**	0.87 (0.76 - 1.01)	0.89 (0.79 - 1.01)

Table A4 Cox regression model for the chance of transplant failure following liver transplantation at night-time compared to daytime

Time from transplant	Unadjusted			Risk adjusted		
	Hazard ratio	95% interval	p-value	Hazard ratio	95% interval	p-value
**30 days**	1.254	1.059 - 1.483	0.009	1.021	0.851 - 1.226	0.8
**One year**	1.247	1.104 - 1.408	0.0004	1.007	0.882 - 1.149	0.9
**Three years**	1.187	1.068 - 1.319	0.002	0.995	0.887 - 1.116	0.9

Table A5 Cox regression model for the chance of graft failure following liver transplantation at night-time compared to daytime

Time from transplant	Unadjusted			Risk adjusted		
	Hazard ratio	95% interval	p-value	Hazard ratio	95% interval	p-value
**30 days**	1.279	1.046 - 1.564	0.02	1.137	0.914 - 1.413	0.2
**One year**	1.227	1.058 - 1.424	0.007	1.071	0.912 - 1.259	0.4
**Three years**	1.233	1.082 - 1.406	0.002	1.112	0.965 - 1.282	0.1

For peer review only

STROBE 2007 (v4) Statement—Checklist of items that should be included in reports of *cohort studies*

Section/Topic	Item #	Recommendation	Reported on page #
**Title and abstract**	1	(*a*) Indicate the study's design with a commonly used term in the title or the abstract	1,2
		(*b*) Provide in the abstract an informative and balanced summary of what was done and what was found	2
**Introduction**			
Background/rationale	2	Explain the scientific background and rationale for the investigation being reported	5
Objectives	3	State specific objectives, including any prespecified hypotheses	6-7
**Methods**			
Study design	4	Present key elements of study design early in the paper	8
Setting	5	Describe the setting, locations, and relevant dates, including periods of recruitment, exposure, follow-up, and data collection	8
Participants	6	(*a*) Give the eligibility criteria, and the sources and methods of selection of participants. Describe methods of follow-up	8
		(*b*) For matched studies, give matching criteria and number of exposed and unexposed	
Variables	7	Clearly define all outcomes, exposures, predictors, potential confounders, and effect modifiers. Give diagnostic criteria, if applicable	9
Data sources/ measurement	8*	For each variable of interest, give sources of data and details of methods of assessment (measurement). Describe comparability of assessment methods if there is more than one group	*8-9*
Bias	9	Describe any efforts to address potential sources of bias	8
Study size	10	Explain how the study size was arrived at10	n/a
Quantitative variables	11	Explain how quantitative variables were handled in the analyses. If applicable, describe which groupings were chosen and why	11-14
Statistical methods	12	(*a*) Describe all statistical methods, including those used to control for confounding	8-9
		(*b*) Describe any methods used to examine subgroups and interactions	7-9
		(*c*) Explain how missing data were addressed	8-9
		(*d*) If applicable, explain how loss to follow-up was addressed	9
		(*e*) Describe any sensitivity analyses	9
**Results**			

For peer review only

Participants	13*	(a) Report numbers of individuals at each stage of study—eg numbers potentially eligible, examined for eligibility, confirmed eligible, included in the study, completing follow-up, and analysed	10
		(b) Give reasons for non-participation at each stage	10
		(c) Consider use of a flow diagram	Na
Descriptive data	14*	(a) Give characteristics of study participants (eg demographic, clinical, social) and information on exposures and potential confounders	10
		(b) Indicate number of participants with missing data for each variable of interest	8, supplementary figure A1
		(c) Summarise follow-up time (eg, average and total amount)	10
Outcome data	15*	Report numbers of outcome events or summary measures over time	10, 11
Main results	16	(*a*) Give unadjusted estimates and, if applicable, confounder-adjusted estimates and their precision (eg, 95% confidence interval). Make clear which confounders were adjusted for and why they were included	11-12
		(*b*) Report category boundaries when continuous variables were categorized	12
		(*c*) If relevant, consider translating estimates of relative risk into absolute risk for a meaningful time period	
Other analyses	17	Report other analyses done—eg analyses of subgroups and interactions, and sensitivity analyses	13-14
**Discussion**			
Key results	18	Summarise key results with reference to study objectives	15
**Limitations**			
Interpretation	20	Give a cautious overall interpretation of results considering objectives, limitations, multiplicity of analyses, results from similar studies, and other relevant evidence	15-17
Generalisability	21	Discuss the generalisability (external validity) of the study results	16-17
**Other information**			
Funding	22	Give the source of funding and the role of the funders for the present study and, if applicable, for the original study on which the present article is based	4

*Give information separately for cases and controls in case-control studies and, if applicable, for exposed and unexposed groups in cohort and cross-sectional studies.

Note: An Explanation and Elaboration article discusses each checklist item and gives methodological background and published examples of transparent reporting. The STROBE checklist is best used in conjunction with this article (freely available on the Web sites of PLoS Medicine at http://www.plosmedicine.org/, Annals of Internal Medicine at http://www.annals.org/, and Epidemiology at http://www.epidem.com/). Information on the STROBE Initiative is available at www.strobe-statement.org.

For peer review only

BMJ Open

Is liver transplantation 'out-of-hours' non-inferior to 'in-hours' transplantation? A retrospective analysis of the UK transplant registry.

Journal:	*BMJ Open*
Manuscript ID	bmjopen-2018-024917.R1
Article Type:	Research
Date Submitted by the Author:	16-Nov-2018
Complete List of Authors:	Halliday, Neil; University College London, Institute of Liver and Digestive Health; University College London, Institute of Immunity and Transplantation<br>Martin, Kate; NHS Blood and Transplant, Statistics and Clinical Studies<br>Collett, David; NHS Blood and Transplant, Statistics and Clinical Audit<br>Allen, Elisa; NHS Blood and Transplant, Statistics and Clinical Studies<br>Thorburn, Douglas; University College London, Institute of Liver and Digestive Health; Royal Free London NHS Trust, Sheila Sherlock Liver Centre
**Primary Subject Heading**:	Gastroenterology and hepatology
Secondary Subject Heading:	Health services research, Surgery
Keywords:	TRANSPLANT MEDICINE, TRANSPLANT SURGERY, Hepatology < INTERNAL MEDICINE, Quality in health care < HEALTH SERVICES ADMINISTRATION & MANAGEMENT

SCHOLARONE™
Manuscripts

Is liver transplantation 'out-of-hours' non-inferior to 'in-hours' transplantation? A retrospective analysis of the UK transplant registry.

Neil Halliday, MBBS[1,3]

Kate Martin, MSc[2]

David Collett, PhD[2]

Elisa Allen, PhD[2]

Douglas Thorburn, MD[1]

1: Sheila Sherlock Liver Centre, Royal Free Hospital and the UCL Institute for Liver and Digestive Health, Pond Street, London

2: Statistics and Clinical Studies, NHS Blood and Transplant, Bristol

3: Institute of Immunity and Transplantation, University College London, London

Corresponding Author: Dr Neil Halliday

neilhalliday@doctors.org.uk

Institute for Liver and Digestive Health

University College London

Upper Third Floor, Royal Free Campus

Rowland Hill Street

London, NW3 2PF

Word count: 3898

For peer review only

Is liver transplantation 'out-of-hours' non-inferior to weekday transplantation? A retrospective analysis of the UK transplant registry.

Objectives: Increased morbidity and mortality have been associated with weekend and night-time clinical activity. We sought to compare the outcomes of liver transplantation (LT) between weekdays and weekends or night-time and day-time to determine if 'out-of-hours' LT has acceptable results compared to 'in-hours'.

Design, setting and participants: We conducted a retrospective analysis of patient outcomes of all 8816 adult, liver-only recipients (2000-2014) from the UK Transplant Registry.

Outcome measures: Outcome measures were graft failure (loss of the graft with or without death) and transplant failure (either graft failure or death with a functioning graft) at 30 days, one-, and three-years post transplantation. The association of these outcomes with weekend vs weekday and day vs night transplantation were explored, following the construction of a risk adjusted Cox regression model.

Results: Similar patient and donor characteristics were observed between weekend and weekday transplantation. Unadjusted graft failure estimates were 5.7% at 30 days, 10.4% at one year and 14.6% at three years; transplant failure estimates were 7.9%, 15.3% and 21.3% respectively.

A risk adjusted Cox regression model demonstrated a significantly lower adjusted hazard ratio (95% CI) of transplant failure for weekend transplant of 0.77 (0.66 - 0.91) within 30 days, 0.86 (0.77 - 0.97) within one year, 0.89 (0.81 - 0.99) within three years and for graft failure of 0.81 (0.67 – 0.97) within 30 days. For patients without transplant failure within 30 days, there was no weekend effect on transplant

For peer review only

failure. Neither night-time procurement nor transplantation were associated with an increased hazard of transplant or graft failure.

Conclusions: Weekend and night-time LT outcomes are non-inferior to weekday or day-time transplantation and we observed a possible small beneficial effect of weekend transplantation. The structure of LT services in the UK delivers acceptable outcomes 'out-of-hours' and may offer wider lessons for weekend working structures.

For peer review only

Article Summary: Strengths and limitations

- This is the first study to address whether there is a weekend effect upon clinical outcomes for liver transplantation in the UK.
- The study was based on an assessment of a large, unbiased, multicentre dataset of all UK liver transplant recipients occurring in the study period.
- The UK transplant registry is a well curated, highly complete database enabling the generation of risk adjusted models including recipient, donor and technical parameters that may influence outcomes.
- Transplantation settings offer the ability to explore outcomes where the timing of clinical event is not determined by the recipient's clinical status
- The major limitations of this study include the inability to identify causative factors, nor identify confounding factors that may be driving differences in outcomes.

Sources of funding: This research received no specific grant from any funding agency in the public, commercial or not-for-profit sectors. NH is supported by a Wellcome Trust PhD Studentship.

Competing interests: The authors declare no competing interests.

Author contributions: NH designed the study concept and strategy, undertook data analysis and drafted and revised the manuscript. KM proposed statistical methods, undertook data analysis and drafted the manuscript. DC proposed statistical methods, reviewed data analysis and drafted the manuscript. EA proposed statistical methods, undertook data analysis and drafted the manuscript. DT designed the study concept and strategy, undertook data analysis and drafted and revised the manuscript.

Data Sharing: Data and statistical code may be made available on request by enquiry via the corresponding author, following approval by NHS Blood and Transplant.

For peer review only

Introduction

Increased morbidity and mortality has been observed with out-of-hours clinical practice in a range of settings[1], which has in part been ascribed to differing clinical service provision through the week. Liver transplantation (LT) services are structured differently to most other clinical services[2] due to the complexity, time sensitivity, scarcity of donations and potential risk of LT. All aspects of LT care are consultant-led, with a standardised service provided at all times and multiple clinical teams including surgeons, anaesthetists, physicians, radiologists and intensive care specialists involved in each case, assisted by specialist co-ordinating staff. Whether this service structure protects against potential weekend effects has not previously been explored in the United Kingdom (UK).

Several studies report excess mortality associated with weekend hospital admission in the UK [3-7] and elsewhere [1 8 9]. However, despite adverse weekend effects being observed in many studies they are not consistent across all diagnoses or presentations and only a proportion of clinical presentations have an observable weekend effect[10-12]. Even within conditions associated with adverse weekend effects, conflicting outcomes have been reported in some [13-15], but not all [16-19] studies and similarly to medical presentation, surgical and intensive care unit (ICU) studies have conflicting results [9 10 20-32]. These findings suggest that adverse weekend effects are complex, disease specific and may have different underlying causes including service structure[19]. Despite this, a recent assessment of the impact of enhanced 7 day working practices in the UK did not show a beneficial impact on adverse weekend outcomes [33].

For peer review only

The current evidence for out-of-hours LT outcomes is mixed. No increased risk of mortality or graft failure was demonstrated with weekend or night-time LT in a multi-centre American study of nearly 95,000 transplants [34]. Another single centre American study demonstrated no increase in surgical complications or long-term mortality but did show an increase in early mortality following LT at night [35]. Renal transplantation at the weekend was not associated with increased mortality or graft failure in a UK study of nearly 13,000 transplants [36], a smaller German study [37] (although higher rates of surgical complications were observed), or a large American study [38]

It remains unclear whether the observed excess mortality associated with weekend admission is a product of differing case severity [39] [40], admission thresholds [41], systematic differences in care delivery, structure and staffing of services, quality of care, poor quality data recording [17] or is an artefact [42]. As weekend effects are specific to different diagnoses and clinical scenarios, if the differences in clinical outcomes are due to service provision and structure, each clinical service structure should be tested for acceptable of outcomes across the week.

The UK delivers LT services with a high volume, low centre-number model, with seven centres providing services for a population of approximately 65 million people, each performing between 30 to 172 deceased donor, adult-recipient LTs annually [43]. With the development of a national organ retrieval service with stipulated retrieval response times and increasing reliance on donation after cardiac death (DCD), the

For peer review only

incidence of out-of-hours transplantation has been increasing. We wanted to establish whether the model of service delivery in the UK ensures consistency in outcomes throughout the day and week. We retrospectively assessed the hazard of graft failure or transplant failure following single organ LT across all UK centres, comparing weekday with weekend and day with night transplantation.

Methods

Data on all adult recipients (≥17 years) of liver only transplants from deceased donors in the UK under the National Health Service (NHS) between 1st January 2000 and 31st December 2014 were obtained from the UK Transplant Registry and followed up to 18th February 2016.

Night-time procurement was deemed to be any liver donation where the liver perfusion start time was between 7pm and 7am. We estimated transplant operation time by adding donor liver perfusion start time (effectively the time of organ retrieval) to cold ischaemia time (CIT) (the time between perfusion and the re-establishment of circulation to the graft, within the recipient). Liver perfusion data was not collected before 2000 and so we have only included transplants since 2000 in the cohort. Night-time transplantation was defined as operation time between 7pm and 7am. Weekend transplantation was defined as any transplant operation time between 5pm on a Friday and 8am on a Monday whereas weekday transplantation included all other time points. These time points were selected to ensure that our findings were comparable to other published studies[34].

For peer review only

The primary outcomes were graft failure and transplant failure. Transplant failure was defined as the earlier of graft failure or patient death (graft failure before death, graft failure and death, or death with a functioning graft were classed as an event), whereas graft failure classed graft failure before death or graft failure and death as an event. Therefore, patients who underwent re-transplantation would have had an event of graft failure associated with their first liver transplant. T-test, chi-square, and log rank tests were used to compare weekday with weekend transplant for continuous, categorical, and failure rate data, respectively. Cox proportional hazards models were built to estimate graft and transplant failure at 30 days, one, and three years post-transplant. Hazard ratios for different time periods were found by including a period factor in the model. Factors considered for inclusion in the model are listed in table 1. Stepwise variable selection, a combination of forwards and backwards selection, was used to identify factors to be included in the models for the different end points guided by a combination of statistical significance and clinical considerations.

Less than 5% of values for each baseline patient, donor and operative characteristic were missing (see supplementary table A1). Missing values for the following recipient factors: international normalised ratio (INR), sodium, creatinine, and bilirubin (used in calculating Model of End-stage Liver Disease (MELD) and United Kingdom model for End-stage Liver Disease (UKELD) score), body mass index (BMI), CIT, on renal support, acute failure grade, in-patient, ventilated, oesophageal varices, sepsis confirmed, previous abdominal surgery; and surgical factors: suboptimal organ appearance, night-time procurement, night-time transplant and weekend transplant were imputed using multiple imputation based on chained

For peer review only

equations [44]. This involved generating 11 data sets with imputed values, with the median of continuous variables and the modal value of the categorical variables being used to produce the final data set. These factors were investigated for any pattern of missingness, but there was generally no evidence of systematic difference in missingness for transplant failure, with the exception of donor type. At one year follow up there were more cases of missing patient or graft outcomes for DCD transplants (14%) compared to donor with brainstem death (DBD) transplants (7.7%).

To assess the fit of the models at each endpoint we used the May and Hosmer test [45]. Schoenfeld residuals and the Grambsch and Therneau test were used to test the assumption of proportional hazards. The functional form of each continuous variable in the model was assessed for non-linearity using martingale residual plots from the null model and by fitting spline terms. The predictive ability of the models was summarised using the c-statistic [46].

Patient involvement: Patients, carers and lay people were not directly involved in the design, conduct and analysis of this study, as it is based on routinely collected data from the UK Transplant Registry. However the study was designed to assess outcomes that are important to patients including graft failure and mortality. The relevance and timeliness of the study was endorsed by the NHS Blood and Transplant Liver Advisory Group, which includes transplant clinicians, patients and lay members

Ethical approval was not required as this study relied solely on retrospective analysis

For peer review only

of pseudanonymised patient data collected for the purposes of clinical care and programme outcome evaluation.

Data supplied to the UK Transplant Registry are validated on receipt to ensure completeness of follow up. Transplant centres are contacted directly if there are validation queries, or to obtain complete data records. Patient survival is confirmed through death registration where possible. All analysis was performed using SAS 9.4 (SAS Institute, Cary NC).

Results

Data were available for 8816 adult LT performed at all UK centres. Follow up information on graft failure or patient death was available at 30 days for all transplants, at one year for 91.4%, and at three years for 76.2% of transplants. Follow up information is obtained from annual follow up appointments with patients, or notification of death. In the analysis, follow up information was censored at the last known follow up for the patient within the follow up period of analysis (30 days, 1 or 3 years post transplant).

The mean recipient age (standard deviation (SD)) at transplantation was 50 (13) years, 60% of the population were male and 88% were Caucasian. Alcohol related liver disease was the leading indication for transplantation (20%) followed by chronic viral hepatitis (16%), acute liver failure (12%), re-transplantation (10%), primary biliary cholangitis (10%), primary sclerosing cholangitis (8%), autoimmune hepatitis (8%), primary liver cancer (6%), metabolic liver disease (6%), and other diagnoses

For peer review only

(5%). The donor population were 51% male with a mean average age (SD) of 46 (16) years. The mean CIT (SD) was 9.3 (3) hours. Eleven percent of livers were from DCDs and the remainder from DBDs.

Of the 8816 transplants, 3203 (36.3%) were performed in the weekend period and 6101 (69.2%) at night. Overall unadjusted transplant failure estimates were 7.9% at 30 days, 15.3% at one year, and 21.3% at three years and graft failure estimates were 5.7%, 10.4%, and 14.6%, respectively.

Effect of weekend transplantation

Table 1 summarises the donor and recipient characteristics by weekday and weekend transplantation, which were all tested for inclusion in the model building process. Transplants at the weekend had a higher frequency of factors associated with poorer outcomes including being an in-patient, being listed for super-urgent indications and having active sepsis at the time of transplantation, however more split liver grafts were performed on weekdays. Lower mean average MELD and UKELD scores were seen on weekdays compared to weekends (19.1 vs 19.5 (p=0.05) and 56.0 vs 56.4 (p=0.02) respectively) although the difference is small and may not be clinically meaningful. Night-time procurement and transplantation were more likely at the weekend.

Similar proportions of first- and re-transplant procedures, organs from DCDs or DBDs and livers from paediatric or adult donors were seen between weekend and weekday recipients.

For peer review only

The following factors were found to be non-significant when comparing weekday and weekend transplants, and in the risk-adjusted models: recipient gender, cause of recipient death, cause of graft failure, donor gender, outcome of first offer (decline/accept).

In the unadjusted analysis, graft failure was similar for weekday and weekend transplantation with 6% and 5% graft failure at 30 days respectively, 11% and 10% at one year, and 15% and 14% at three years. Transplant failure was higher amongst weekday recipients at 30 days (8% vs 7% (p=0.01)), but not significantly different at other time points (see Figure 1).

The factors that significantly affected transplant failure at each of the three time points and were included in each model were: recipient factors: on renal support, ventilated, confirmed sepsis at time of transplantation, primary liver disease, age, in-patient at time of transplantation, acute liver failure, previous abdominal surgery, presence of oesophageal varices, presence of TIPS, Caucasian: graft factors: organ appearance suboptimal, CIT, donor age, DCD, split liver and transplant year and night-time transplant. Each model was built separately for the different endpoints and outcomes (graft failure or transplant failure) and any significant factors were included in the risk-adjusted models. Supplementary Tables A2 and A3 demonstrate the risk adjusted hazard ratio for each variable from the Cox regression model for the chance of transplant failure or graft failure, respectively. The c-statistics for the transplant failure and graft failure models were 0.65, 0.63, and 0.60; and 0.64, 0.62 and 0.60 at 30 days, 1 year, and 3 years, respectively.

For peer review only

The risk adjusted hazard ratio (95% confidence interval) of transplant failure for weekend transplant relative to weekday was 0.77 (0.66 - 0.91) within 30 days. The corresponding hazard ratios for one year and three years were 0.86 (0.77 - 0.97), 0.89 (0.81 - 0.99), respectively (table 2). A weekend transplant had a significant effect on hazard of graft failure alone at 30 days post-transplant of 0.81 (0.67 – 0.97), and a marginal at one and three years post-transplant (table 3). To ensure that the imputed data did not influence the outcomes, analysis excluding the cases with imputed data (therefore including 8037 cases) revealed a similar pattern of results (data not shown).

Differences in surgical complexity could potentially influence outcomes between time periods, for example if more complex patients were selected for transplantation during the week. There is no direct measure of surgical complexity available, but factors that may reflect this were similar between weekdays and weekends including the mean number of units of blood transfused intra-operatively (5.1 weekdays vs 5.0 weekends (p=0.6)), mean length of in-patient stay (21.9 days and 22.4 days respectively (p=0.24)), presence of portal vein thrombosis (2% vs 2% (p=0.9)), recipient BMI (26.4 vs 26.4kg/m^2 (p=0.5)), presence of TIPS (3% vs 4% (p=0.06)) and prior abdominal surgery (20% vs 21% (p=0.2) although the mean length of ICU stay was longer with weekend transplantation (5.4 (SD 9) vs 6.1 (SD 12) days (p=0.008)).

To explore whether there were specific periods in the week that were associated with increased risk of transplant or graft failure, we tested early and late weekdays and each day individually. There was no observed difference in graft or transplant failure

between patients transplanted during the day in the early week (Monday 8am-7pm, Tuesday and Wednesday 7am-7pm) and the late week (Thursday 7am-7pm and Friday 7am-5pm), and the day of the week did not significantly affect the risk of failure, after risk-adjustment, at any of the three time points. An interaction between weekend and night-time transplant was found to be non-significant (p=0.9 at 30 days, 0.5 at 1 year and 0.7 at 3 years). As there were large differences in the number of transplants performed per centre during the study period (range 533 to 2112) we tested whether there were variations in the frequency of weekend transplantation and in weekend outcomes on a per centre basis. The weekend effect was consistent across all transplant centres, and an interaction between weekend transplant and centre was non-significant (p=0.3 at 30 days, 0.2 at 1 year and 0.1 at 3 years).

We considered whether there were different rates of higher risk organ use between weekends and weekdays. We observed that use of organs previously declined by another centre at their first offering was the same for weekdays (31%) and weekends (32%) (p=0.23) and the proportion declined due to donor factors, as compared to other factors, was identical. Donor age, use of DCDs, CIT and suboptimal organ appearance were similar.

To determine when the factors leading to reduced outcomes following weekday transplantation were operating, analysis was restricted to those who did not suffer death or graft failure prior to 30 days. This resulted in no significant weekend effect on transplant failure after one and three years, indicating that the factors operate

For peer review only

within the first month following transplantation and continue to effect long term failure rates.

Effect of night-time procurement or transplantation

A similar analysis and model building strategy was undertaken for transplants assessed as day-time (operative start time 7am-7pm) compared to night-time and also for day-time compared to night-time organ procurement. 2715 (31%) transplants were undertaken during the day-time and 6101 (69%) at night. Night-time procurement occurred in 6228 (71%) of transplants. Day-time compared to night-time transplant was associated with lower graft failure at 30 days (5% vs 6% (p=0.02)), one year (9% vs 11% (p=0.007)), and three years (13% vs 15% (p=0.002)) and transplant failure at 30 days (7% vs 8% (p=0.008)) and one year (13% vs 16% (p=0.0004)), and three years (19% vs 22% (p=0.001)) in the unadjusted analysis. Day-time compared to night-time procurement was associated with lower graft failure at 30 days (5% vs 6% (p=0.04)), one year (9% vs 11% (p=0.005)), and three years (13% vs 15% (p=0.002)) and transplant failure at 30 days (7% vs 8% (p=0.03)), one year (13% vs 16% (p=0.0007)), and three years (19% vs 22% (p=0.003)) in the unadjusted analysis. In the Cox proportional hazard models, utilising the same variables as for the weekend vs weekday model neither night-time procurement nor night-time transplantation had a significant effect on transplant or graft failure at any time point (Supplementary Tables A4 and A5).

For peer review only

Discussion

We have demonstrated no increased risk of graft or transplant failure with weekend LT, night-time LT or night-time graft procurement. These findings suggest that UK liver transplant outcomes do not have an increased risk of adverse outcomes associated with 'out-of-hours' operating. Interestingly there is a possible reduction in the hazard of early graft failure and long-term transplant failure associated with weekend transplantation. The loss of this association when considering only survivors at 30 days suggests that any responsible factors are acting in the peri- and early post-transplant period. The effect is present in the unadjusted transplant failure data at 30 days, in the adjusted outcomes at all time points measured, and is seen across all centres.

There is a wide range of putative confounding factors that may influence the risk of graft and transplant failure during in-hours and out-of-hours transplantation. We have attempted to control for those that were measured in the database including donor, operative and recipient characteristics (as listed in table 1). Unmeasured or non-quantifiable confounders such as risk aversion in operator practice, clinical team structure or seniority, pressure on general hospital resources, donor graft quality and patient fitness may still be operating, potentially confounding our observations. As explained below we have attempted to identify proxies for these where possible but due to the complex and multifactorial influences on liver transplant outcomes we cannot exclude the presence of confounding effects.

To explore whether the finding of possible improved weekend outcomes could be explained we tested several hypotheses. Patients transplanted at weekends were

For peer review only

not lower risk; in fact they were more likely to have adverse prognostic markers and there were no differences in the causes of liver disease between the two groups. There were no systematic differences in organ utilisation: markers of adverse graft features were similar including average donor ages and the proportion of DCD, suboptimal and previously declined organs that were transplanted. No differences in markers of surgical complexity were noted including portal vein thrombosis, oesophageal varices, re-transplantation, prior abdominal surgery, BMI, and volume of blood transfused intraoperatively. There was no evidence for systemic delaying of operative start times following organ retrieval, as the CIT was similar. If this were occurring to ensure adequate practitioner rest at night or prioritise elective commitments, it would theoretically be seen more in weekday transplantation.

A lead in effect, where patients transplanted in the week are stepped down from the ICU to general wards over the weekend, would disproportionally affect transplants occurring early in the week, however we found no such association. Weekend LT was associated with a longer average ICU stay (6.1 vs 5.4 days) but whether this would improve outcomes, was a marker of a more unwell patient population, or is of any clinical importance is unclear. Therefore we are unable to explain this association and it may relate to other unmeasured factors in patient or graft selection or care.

We speculate that the senior-clinician led and delivered service delivery may obviate any inherent weekend risks. Although as previously noted, not all clinical scenarios are associated with 'out-of-hours' risks. However this service structure would also not explain difference we observed between weekend and weekday outcomes. It is

For peer review only - http://bmjopen.bmj.com/site/about/guidelines.xhtml

conceivable that there may be a protective effect of weekend staffing patterns or hospital environment upon LT outcomes, for example, does the absence of routine elective work and competing clinical activity potentially free clinicians and resources for a more prompt and responsive service?

We have demonstrated no increased hazard of graft or transplant failure for liver transplants performed out-of-hours. Whether this is due to weekends being inherently risky (due to e.g. operator fatigue, reduced staffing and ancillary support services) but that the senior led service and lack of competing clinical activity prevents this from leading to poor patient outcomes, or conversely that in- and in out-of-hours transplantation carry a similar baseline risk cannot be unpicked in this study. If the inverse weekend effect we observe is true, we believe the modifying factor likely lies in differences in staffing and competing clinical activity as outlined above.

The limitations of this work include that, as with all retrospective registry based studies, neither causality nor aetiological factors can be identified. Furthermore registry data is dependent upon the quality of data imputing, curation, and assignation of variables. However, this is a well curated, highly complete dataset that has previously been used for multiple studies. The definition of day and night in regard to transplantation surgery is artificial as combined organ retrieval, transfer, and implantation straddles both day and night periods but our methodology has been utilised in a previous similar study of LT [34]. Due to the large size of the database some observed differences will reach statistical significance, despite being clinically insignificant (for example, the statistically significant but very small difference

For peer review only

observed in UKELD score between weekday and weekend transplantation groups) and as such differences should be interpreted with caution. Likewise, due to the modest difference in graft and transplant survival observed between these two groups we suggest that there at least is no evidence for worse outcomes at the weekend.

This study is an interesting comparator for published disease specific and unselected admissions studies of out-of-hours outcomes [3-17 20-24 27 28]. Our cohort of patients had a standardised risk profile throughout the week unlike unselected admission studies, as to qualify for LT they had to meet minimum clinical thresholds, assessed by objective validated clinical scores, yet had to be well enough for the procedure to be performed, thus obviating the selection bias inherent in hospital admission studies. Furthermore the availability of an organ rather than a patient's clinical status determined the timing of the admission and clinical intervention, in contrast to unselected admission studies. Finally our study benefited from a detailed, well-curated database of individual patient clinical parameters and outcomes that enabled accurate risk adjustment models to correct for variation in clinical risk on a per patient basis.

The non-inferiority of out-of-hours LT, as seen in other LT and renal transplant studies [34-38], is reassuring and illustrates that the current model of liver transplant provision in the UK provides acceptable outcomes at traditionally perceived periods of clinical risk. However, the potential for improved weekend clinical outcomes differs to other studies on LT [34 35]. A potential beneficial weekend effect is interesting as if working patterns or hospital resources are responsible this represents a model for

For peer review only

improved patient care. Direct comparison of surgeons', ICU, and physicians' workload, clinical commitments, and working patterns between weekdays and weekends and comparisons with other, senior-clinician led services will be of interest.

In summary, we have demonstrated that 'out-of-hours' LT outcomes are not worse than for 'in-hours' procedures and potentially weekend liver transplantation may be associated with reduced adverse outcomes. The weekend LT care structure in the UK may represent a model for the design of other critical out-of-hours services. Furthermore these findings illustrate the complexity of observed weekend effects, which are likely to be dependent on patient selection.

References

1. Hoshijima H, Takeuchi R, Mihara T, et al. Weekend versus weekday admission and short-term mortality: A meta-analysis of 88 cohort studies including 56,934,649 participants. *Medicine* 2017;96(17):e6685. doi: 10.1097/MD.0000000000006685
2. England N. https://www.england.nhs.uk/publication/liver-transplantation-service-adults/ (accessed 9th April 2018).
3. Freemantle N, Richardson M, Wood J, et al. Weekend hospitalization and additional risk of death: an analysis of inpatient data. *Journal of the Royal Society of Medicine* 2012;105(2):74-84. doi: 10.1258/jrsm.2012.120009 [published Online First: 2012/02/07]
4. Freemantle N, Ray D, McNulty D, et al. Increased mortality associated with weekend hospital admission: a case for expanded seven day services? *BMJ (Clinical research ed)* 2015;351:h4596. doi: 10.1136/bmj.h4596 [published Online First: 2015/09/08]
5. Aylin P, Yunus A, Bottle A, et al. Weekend mortality for emergency admissions. A large, multicentre study. *Quality & safety in health care* 2010;19(3):213-7. doi: 10.1136/qshc.2008.028639 [published Online First: 2010/01/30]
6. Handel AE, Patel SV, Skingsley A, et al. Weekend admissions as an independent predictor of mortality: an analysis of Scottish hospital admissions. *BMJ open* 2012;2(6) doi: 10.1136/bmjopen-2012-001789 [published Online First: 2012/11/09]
7. Mohammed MA, Sidhu KS, Rudge G, et al. Weekend admission to hospital has a higher risk of death in the elective setting than in the emergency setting: a retrospective database study of national health service hospitals in England. *BMC health services research* 2012;12:87. doi: 10.1186/1472-6963-12-87 [published Online First: 2012/04/05]

For peer review only - http://bmjopen.bmj.com/site/about/guidelines.xhtml

8. Barba R, Losa JE, Velasco M, et al. Mortality among adult patients admitted to the hospital on weekends. *European journal of internal medicine* 2006;17(5):322-4. doi: 10.1016/j.ejim.2006.01.003 [published Online First: 2006/07/26]
9. Ruiz M, Bottle A, Aylin PP. The Global Comparators project: international comparison of 30-day in-hospital mortality by day of the week. *BMJ quality & safety* 2015;24(8):492-504. doi: 10.1136/bmjqs-2014-003467 [published Online First: 2015/07/08]
10. Bell CM, Redelmeier DA. Mortality among patients admitted to hospitals on weekends as compared with weekdays. *The New England journal of medicine* 2001;345(9):663-8. doi: 10.1056/NEJMsa003376 [published Online First: 2001/09/08]
11. Zhou Y, Li W, Herath C, et al. Off-Hour Admission and Mortality Risk for 28 Specific Diseases: A Systematic Review and Meta-Analysis of 251 Cohorts. *Journal of the American Heart Association* 2016;5(3):e003102. doi: 10.1161/jaha.115.003102 [published Online First: 2016/03/20]
12. Concha OP, Gallego B, Hillman K, et al. Do variations in hospital mortality patterns after weekend admission reflect reduced quality of care or different patient cohorts? A population-based study. *BMJ quality & safety* 2014;23(3):215-22. doi: 10.1136/bmjqs-2013-002218 [published Online First: 2013/10/29]
13. Ananthakrishnan AN, McGinley EL, Saeian K. Outcomes of weekend admissions for upper gastrointestinal hemorrhage: a nationwide analysis. *Clinical gastroenterology and hepatology : the official clinical practice journal of the American Gastroenterological Association* 2009;7(3):296-302e1. doi: 10.1016/j.cgh.2008.08.013 [published Online First: 2008/12/17]
14. Shaheen AA, Kaplan GG, Myers RP. Weekend versus weekday admission and mortality from gastrointestinal hemorrhage caused by peptic ulcer disease. *Clinical gastroenterology and hepatology : the official clinical practice journal of the American Gastroenterological Association* 2009;7(3):303-10. doi: 10.1016/j.cgh.2008.08.033 [published Online First: 2008/10/14]
15. Roberts SE, Thorne K, Akbari A, et al. Mortality following Stroke, the Weekend Effect and Related Factors: Record Linkage Study. *PLoS One* 2015;10(6):e0131836. doi: 10.1371/journal.pone.0131836 [published Online First: 2015/06/30]
16. Jairath V, Kahan BC, Logan RF, et al. Mortality from acute upper gastrointestinal bleeding in the United kingdom: does it display a "weekend effect"? *Am J Gastroenterol* 2011;106(9):1621-8. doi: 10.1038/ajg.2011.172 [published Online First: 2011/05/25]
17. Li L, Rothwell PM. Biases in detection of apparent "weekend effect" on outcome with administrative coding data: population based study of stroke. *BMJ (Clinical research ed)* 2016;353:i2648. doi: 10.1136/bmj.i2648 [published Online First: 2016/05/18]
18. Murray IA, Dalton HR, Stanley AJ, et al. International prospective observational study of upper gastrointestinal haemorrhage: Does weekend admission affect outcome? *United European gastroenterology journal* 2017;5(8):1082-89. doi: 10.1177/2050640617700984 [published Online First: 2017/12/15]
19. Gupta A, Agarwal R, Ananthakrishnan AN. "Weekend Effect" in Patients With Upper Gastrointestinal Hemorrhage: A Systematic Review and Meta-analysis. *Am J Gastroenterol* 2017 doi: 10.1038/ajg.2017.430 [published Online First: 2017/11/15]
20. Aylin P, Alexandrescu R, Jen MH, et al. Day of week of procedure and 30 day mortality for elective surgery: retrospective analysis of hospital episode statistics. *BMJ (Clinical research ed)* 2013;346:f2424. doi: 10.1136/bmj.f2424 [published Online First: 2013/05/30]

For peer review only

21. McIsaac DI, Bryson GL, van Walraven C. Elective, major noncardiac surgery on the weekend: a population-based cohort study of 30-day mortality. *Medical care* 2014;52(6):557-64. doi: 10.1097/mlr.0000000000000137 [published Online First: 2014/05/03]
22. Zapf MA, Kothari AN, Markossian T, et al. The "weekend effect" in urgent general operative procedures. *Surgery* 2015;158(2):508-14. doi: 10.1016/j.surg.2015.02.024 [published Online First: 2015/05/28]
23. Groves EM, Khoshchehreh M, Le C, et al. Effects of weekend admission on the outcomes and management of ruptured aortic aneurysms. *Journal of vascular surgery* 2014;60(2):318-24. doi: 10.1016/j.jvs.2014.02.052 [published Online First: 2014/04/09]
24. Goldstein SD, Papandria DJ, Aboagye J, et al. The "weekend effect" in pediatric surgery - increased mortality for children undergoing urgent surgery during the weekend. *Journal of pediatric surgery* 2014;49(7):1087-91. doi: 10.1016/j.jpedsurg.2014.01.001 [published Online First: 2014/06/24]
25. Metcalfe D, Castillo-Angeles M, Rios-Diaz AJ, et al. Is there a "weekend effect" in emergency general surgery? *The Journal of surgical research* 2018;222:219-24. doi: 10.1016/j.jss.2017.10.019 [published Online First: 2017/12/24]
26. Smith SA, Yamamoto JM, Roberts DJ, et al. Weekend Surgical Care and Postoperative Mortality: A Systematic Review and Meta-Analysis of Cohort Studies. *Medical care* 2017 doi: 10.1097/mlr.0000000000000860 [published Online First: 2017/12/19]
27. Knudsen NV, Moller MH. Association of mortality with out-of-hours admission in patients with perforated peptic ulcer. *Acta anaesthesiologica Scandinavica* 2015;59(2):248-54. doi: 10.1111/aas.12450 [published Online First: 2014/12/17]
28. McVay DP, Walker AS, Nelson DW, et al. The weekend effect: does time of admission impact management and outcomes of small bowel obstruction? *Gastroenterology report* 2014;2(3):221-5. doi: 10.1093/gastro/gou043 [published Online First: 2014/07/11]
29. Barnett MJ, Kaboli PJ, Sirio CA, et al. Day of the week of intensive care admission and patient outcomes: a multisite regional evaluation. *Medical care* 2002;40(6):530-9. [published Online First: 2002/05/22]
30. Cavallazzi R, Marik PE, Hirani A, et al. Association between time of admission to the ICU and mortality: a systematic review and metaanalysis. *Chest* 2010;138(1):68-75. doi: 10.1378/chest.09-3018 [published Online First: 2010/04/27]
31. Galloway M, Hegarty A, McGill S, et al. The Effect of ICU Out-of-Hours Admission on Mortality: A Systematic Review and Meta-Analysis. *Critical care medicine* 2017 doi: 10.1097/ccm.0000000000002837 [published Online First: 2017/11/15]
32. Arabi Y, Alshimemeri A, Taher S. Weekend and weeknight admissions have the same outcome of weekday admissions to an intensive care unit with onsite intensivist coverage. *Critical care medicine* 2006;34(3):605-11. [published Online First: 2006/03/08]
33. Meacock R, Sutton M. Elevated mortality among weekend hospital admissions is not associated with adoption of seven day clinical standards. *Emergency medicine journal : EMJ* 2017 doi: 10.1136/emermed-2017-206740 [published Online First: 2017/11/10]
34. Orman ES, Hayashi PH, Dellon ES, et al. Impact of nighttime and weekend liver transplants on graft and patient outcomes. *Liver Transpl* 2012;18(5):558-65. doi: 10.1002/lt.23395 [published Online First: 2012/01/25]

For peer review only

35. Lonze BE, Parsikia A, Feyssa EL, et al. Operative start times and complications after liver transplantation. *Am J Transplant* 2010;10(8):1842-9. doi: 10.1111/j.1600-6143.2010.03177.x [published Online First: 2010/07/28]
36. Anderson BM, Mytton JL, Evison F, et al. Outcomes After Weekend Admission for Deceased Donor Kidney Transplantation: A Population Cohort Study. *Transplantation* 2017;101(9):2244-52. doi: 10.1097/tp.0000000000001522 [published Online First: 2016/10/22]
37. Schutte-Nutgen K, Tholking G, Dahmen M, et al. Is there a "weekend effect" in kidney transplantation? *PLoS One* 2017;12(12):e0190227. doi: 10.1371/journal.pone.0190227 [published Online First: 2017/12/29]
38. Baid-Agrawal S, Martus P, Feldman H, et al. Weekend versus weekday transplant surgery and outcomes after kidney transplantation in the USA: a retrospective national database analysis. *BMJ open* 2016;6(4):e010482. doi: 10.1136/bmjopen-2015-010482 [published Online First: 2016/04/09]
39. Anselmi L, Meacock R, Kristensen SR, et al. Arrival by ambulance explains variation in mortality by time of admission: retrospective study of admissions to hospital following emergency department attendance in England. *BMJ quality & safety* 2017;26(8):613-21. doi: 10.1136/bmjqs-2016-005680 [published Online First: 2016/11/01]
40. Walker AS, Mason A, Quan TP, et al. Mortality risks associated with emergency admissions during weekends and public holidays: an analysis of electronic health records. *Lancet (London, England)* 2017;390(10089):62-72. doi: 10.1016/s0140-6736(17)30782-1 [published Online First: 2017/05/14]
41. Meacock R, Anselmi L, Kristensen SR, et al. Higher mortality rates amongst emergency patients admitted to hospital at weekends reflect a lower probability of admission. *Journal of health services research & policy* 2017;22(1):12-19. doi: 10.1177/1355819616649630 [published Online First: 2016/06/04]
42. Bray BD, Steventon A. What have we learnt after 15 years of research into the 'weekend effect'? *BMJ quality & safety* 2017;26(8):607-10. doi: 10.1136/bmjqs-2016-005793 [published Online First: 2016/12/03]
43. Martin K, Allen E, Johnson R, et al. Annual report on liver transplantation: report for 2014/2015. 2015. http://www.odt.nhs.uk/pdf/organ_specific_report_liver_2015.pdf (accessed June 2016).
44. White IR, Royston P, Wood AM. Multiple imputation using chained equations: Issues and guidance for practice. *Statistics in medicine* 2011;30(4):377-99. doi: 10.1002/sim.4067 [published Online First: 2011/01/13]
45. May S, Hosmer DW. A simplified method of calculating an overall goodness-of-fit test for the Cox proportional hazards model. *Lifetime data analysis* 1998;4(2):109-20. [published Online First: 1998/07/11]
46. Collett D. Modelling survival data in medical research. London: Chapman & Hall 2015.

For peer review only

Tables

Table 1 Characteristics of weekday and weekend liver only transplants in the UK, 1st January 2000 to 31st December 2014

	Weekday transplant	Weekend transplant	p-value
	N (%) unless otherwise stated		
**Total number of transplants**	**5613 (64)**	**3203 (36)**	
**Recipient characteristics**			
Transplant year			
2000	312 (6)	209 (7)	
2001	345 (6)	188 (6)	
2002	335 (6)	232 (7)	
2003	288 (5)	207 (6)	
2004	340 (6)	239 (7)	
2005	315 (6)	172 (5)	
2006	321 (6)	196 (6)	0.002
2007	341 (6)	198 (6)	
2008	379 (7)	195 (6)	
2009	379 (7)	187 (6)	
2010	379 (7)	212 (7)	
2011	406 (7)	209 (7)	
2012	441 (8)	227 (7)	
2013	511 (9)	246 (8)	
2014	521 (9)	286 (9)	
Super - urgent	803 (14)	523 (16)	0.01
Age at transplant, mean (SD)	50 (13)	50.2 (12)	0.5
Male gender	3405 (61)	1899 (59)	0.2
Caucasian	4922 (88)	2814 (88)	0.8
MELD at transplant, mean (SD)	19.1 (9)	19.5 (9)	0.05
UKELD at transplant, mean (SD)	56.0 (6)	56.4 (7)	0.02
Primary liver disease			
Cancer	320 (6)	171 (5)	
HCV	746 (13)	395 (12)	
Alcohol related liver disease	1180 (21)	626 (20)	
HBV	153 (3)	107 (3)	
PSC	451 (8)	276 (9)	0.14
PBC	520 (9)	333 (10)	
Autoimmune hepatitis	453 (8)	255 (8)	
Metabolic liver disease	316 (6)	172 (5)	
Acute liver disease	647 (12)	414 (13)	
Re-transplants	539 (10)	304 (9)	
Other	288 (5)	150 (5)	
ABO Blood group			
O	2405 (43)	1318 (41)	
A	2338 (42)	1341 (42)	0.01
B	648 (12)	372 (12)	
AB	222 (4)	172 (5)	
Renal support	690 (12)	421 (13)	0.2
In-patient	1585 (28)	975 (30)	0.03
Ventilated	585 (10)	373 (12)	0.08
Oesophageal varices	3406 (61)	1991 (62)	0.17
Presence of TIPS	185 (3)	130 (4)	0.06

For peer review only

Sepsis confirmed	224 (4)	175 (5)	0.001
Portal vein thrombosis	104 (2)	61 (2)	0.9
BMI kg/m^2, mean (SD)	26.4 (5)	26.4 (5)	0.5
**Donor characteristics**			
Donor age, mean (SD)	46.4 (15)	46 (16)	0.3
DCD	596 (11)	333 (10)	0.7
Split liver	398 (7)	190 (6)	0.04
Organ appearance suboptimal	1208 (22)	653 (20)	0.2
Cause of death			
Cerebrovascular Accident (CVA)	3736 (67)	2174 (68)	
Miscellaneous	1186 (21)	572 (18)	0.0002
Road Traffic Accident (RTA)	410 (7)	293 (9)	
Other trauma	281 (5)	164 (5)	
**Operative characteristics**			
Night-time procurement	3799 (68)	2429 (76)	<0.0001
Night-time transplant	3715 (66)	2386 (74)	<0.0001
CIT (hours), mean (SD)	9.3 (3)	9.4 (3)	0.24
Previous abdominal surgery	1115 (20)	670 (21)	0.2
**Failure**			
Overall graft failure (%)			
30 day	6	5	0.08
One year	11	10	0.16
Three years	15	14	0.16
Overall transplant failure (%)			
30 day	8	7	0.01
One year	16	14	0.09
Three years	22	20	0.12
**Cause of death at 30 days**	**229 (4)**	**120 (4)**	
Cardiothoracic / Myocardial ischaemia and infarction	29 (13)	17 (14)	
Cerebrovascular accident	11 (5)	2 (2)	
Haemorrhage	15 (7)	7 (6)	0.3
Infection / Septicaemia	32 (14)	15 (13)	
Multi-system failure	67 (29)	47 (39)	
Other	75 (33)	32 (27)	
**Cause of graft failure at 30 days**	**314 (6)**	**152 (5)**	
Biliary complications	6 (2)	4 (3)	
Hepatic artery thrombosis	95 (30)	42 (28)	
Non-thrombotic infarction	14 (4)	12 (8)	0.6
Primary non-function	93 (30)	50 (33)	
Rejection	7 (2)	3 (2)	
Other	99 (32)	41 (27)	

For peer review only

Table 2 **Cox regression model for chance of transplant failure following liver transplantation at the weekend compared to a weekday.**

Time from transplant	Unadjusted			Risk adjusted		
	Hazard ratio	95% interval	p-value	Hazard ratio	95% interval	p-value
**30 days**	0.818	0.698 - 0.959	0.01	0.772	0.658 - 0.906	0.001
**One year**	0.906	0.809 – 1.014	0.09	0.864	0.771 - 0.968	0.01
**Three years**	0.925	0.839 – 1.020	0.12	0.893	0.809 - 0.986	0.02

For peer review only

Table 3 **Cox regression model for chance of graft failure following liver transplantation at the weekend compared to a weekday.**

Time from transplant	Unadjusted			Risk adjusted		
	Hazard ratio	95% interval	p-value	Hazard ratio	95% interval	p-value
**30 days**	0.845	0.700 – 1.020	0.08	0.805	0.665 – 0.973	0.02
**One year**	0.905	0.787 – 1.039	0.16	0.874	0.760 – 1.005	0.06
**Three years**	0.918	0.814 – 1.036	0.16	0.892	0.790 – 1.007	0.06

For peer review only

Figure Legend

Figure 1 Unadjusted transplant failure up to three years post-transplant

For peer review only

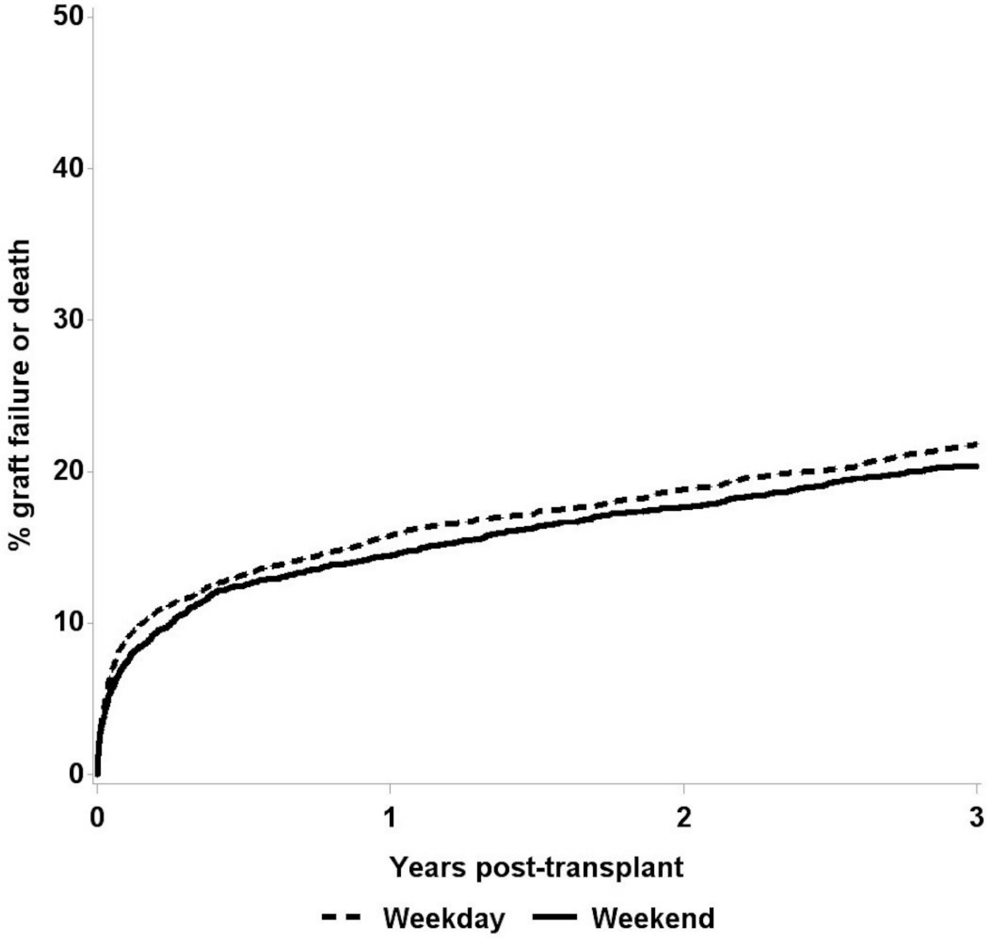

Figure 1 Unadjusted transplant failure up to three years post-transplant

90x90mm (300 x 300 DPI)

Appendix/Supplementary tables

Table A1 Percentage of missing values for each variable of interest, for liver only transplants in the UK, 1st January 2000 to 31st December 2014

**Total number of transplants**	**8816**
	**% missing**
**Recipient characteristics**	
Transplant year	0
Super - urgent	0
Age at transplant, mean (SD)	0
Male gender	0
Caucasian	0
MELD at transplant, mean (SD)	3.2
UKELD at transplant, mean (SD)	3.2
Primary liver disease	0
ABO Blood group	0
Renal support	0.2
In-patient	0.1
Ventilated	0.1
Oesophageal varices	0.4
Presence of TIPS	0
Sepsis confirmed	0.3
Portal vein thrombosis	0
BMI kg/m^2, mean (SD)	3.5
**Donor characteristics**	
Donor age, mean (SD)	0
DCD	0
Split liver	0
Organ appearance suboptimal	0.2
Cause of death	0
**Operative characteristics**	
Night-time procurement	2.3
Night-time transplant	2.5
Weekend transplant	0
CIT (hours), mean (SD)	4.7
Previous abdominal surgery	0.3

For peer review only

Table A2 Risk adjusted hazard ratio from Cox regression model for chance of graft failure or death following liver transplantation in the UK, 1st January 2000 to 31st December 2014

Risk factor	Hazard ratio (95% confidence interval) 30 days	1 year	3 years
**Recipient characteristics**			
Transplant year			
2000	1.00 (-)	1.00 (-)	1.00 (-)
2001	1.07 (0.74 - 1.54)	0.94 (0.71 - 1.23)	0.90 (0.71 - 1.14)
2002	0.82 (0.56 - 1.21)	0.76 (0.58 - 1.01)	0.79 (0.62 - 1.01)
2003	0.92 (0.62 - 1.35)	0.90 (0.68 - 1.18)	0.84 (0.66 - 1.07)
2004	0.92 (0.63 - 1.35)	0.96 (0.74 - 1.25)	0.88 (0.70 - 1.11)
2005	0.75 (0.49 - 1.13)	**0.73 (0.54 - 0.98)**	**0.72 (0.56 - 0.93)**
2006	0.75 (0.50 - 1.12)	0.76 (0.58 - 1.02)	**0.75 (0.59 - 0.96)**
2007	0.71 (0.48 - 1.06)	**0.64 (0.48 - 0.86)**	**0.63 (0.49 - 0.81)**
2008	**0.59 (0.38 - 0.89)**	**0.62 (0.46 - 0.82)**	**0.63 (0.49 - 0.80)**
2009	**0.58 (0.38 - 0.88)**	**0.58 (0.43 - 0.78)**	**0.59 (0.46 - 0.76)**
2010	**0.64 (0.42 - 0.96)**	**0.57 (0.42 - 0.76)**	**0.56 (0.43 - 0.72)**
2011	**0.58 (0.38 - 0.88)**	**0.46 (0.33 - 0.62)**	**0.50 (0.38 - 0.65)**
2012	**0.50 (0.32 - 0.76)**	**0.45 (0.33 - 0.62)**	**0.51 (0.39 - 0.66)**
2013	**0.62 (0.42 - 0.92)**	**0.51 (0.39 - 0.69)**	**0.50 (0.38 - 0.65)**
2014	**0.48 (0.31 - 0.73)**	**0.44 (0.33 - 0.60)**	**0.46 (0.34 - 0.60)**
Age at transplant	**1.01 (1.00 - 1.01)**	**1.01 (1.01 - 1.01)**	**1.01 (1.00 - 1.01)**
Caucasian	0.89 (0.71 - 1.11)	0.85 (0.72 - 1.01)	**0.85 (0.74 - 0.97)**
Primary liver disease			
Cancer	1.00 (-)	1.00 (-)	1.00 (-)
HCV	0.79 (0.50 - 1.24)	0.87 (0.65 - 1.17)	0.94 (0.74 - 1.19)
Alcohol related liver disease	1.05 (0.69 - 1.59)	0.89 (0.67 - 1.18)	**0.76 (0.61 - 0.96)**
HBV	1.00 (0.55 - 1.82)	0.96 (0.64 - 1.44)	0.83 (0.59 - 1.17)
PSC	1.17 (0.73 - 1.85)	1.16 (0.85 - 1.59)	1.03 (0.80 - 1.33)
PBC	0.90 (0.57 - 1.44)	0.84 (0.61 - 1.14)	**0.65 (0.50 - 0.84)**
Autoimmune hepatitis	1.06 (0.66 - 1.70)	1.00 (0.73 - 1.38)	0.85 (0.65 - 1.11)
Metabolic liver disease	1.24 (0.75 - 2.05)	1.14 (0.81 - 1.61)	0.91 (0.68 - 1.23)
Acute liver disease	1.56 (0.91 - 2.67)	1.34 (0.91 - 1.96)	1.07 (0.77 - 1.49)
Re-transplants	**1.77 (1.08 - 2.91)**	**1.74 (1.24 - 2.44)**	**1.52 (1.14 - 2.02)**
Other	0.95 (0.55 - 1.65)	1.04 (0.72 - 1.49)	0.83 (0.61 - 1.13)
Renal support	**1.42 (1.13 - 1.80)**	**1.44 (1.21 - 1.71)**	**1.42 (1.22 - 1.67)**
In-patient	1.08 (0.85 - 1.36)	**1.29 (1.10 - 1.50)**	**1.22 (1.07 - 1.40)**
Ventilated	**1.85 (1.36 - 2.51)**	**1.59 (1.26 - 2.01)**	**1.42 (1.14 - 1.76)**
Oesophageal varices	**1.18 (1.00 - 1.39)**	1.09 (0.96 - 1.23)	1.03 (0.93 - 1.14)
Presence of TIPS	1.08 (0.71 - 1.64)	1.20 (0.91 - 1.60)	1.22 (0.96 - 1.56)
Sepsis confirmed	**1.49 (1.14 - 1.95)**	**1.33 (1.08 - 1.63)**	**1.29 (1.07 - 1.56)**
Acute failure grade prior to transplant	1.06 (0.74 - 1.51)	0.81 (0.62 - 1.05)	0.78 (0.61 - 0.99)
**Donor characteristics**			
Donor age (years)	1.00 (0.99 - 1.01)	**1.01 (1.00 - 1.01)**	**1.01 (1.00 - 1.01)**
DCD	**2.21 (1.69 - 2.88)**	**2.00 (1.65 - 2.43)**	**1.84 (1.55 - 2.17)**
Split liver	1.18 (0.73 - 1.93)	**2.12 (1.63 - 2.76)**	**2.01 (1.59 - 2.53)**
Organ appearance suboptimal	**2.32 (1.80 - 2.98)**	**1.64 (1.39 - 1.92)**	**1.48 (1.29 - 1.70)**

For peer review only

Split liver * time (days post-transplant)	**1.05 (1.01 - 1.08)**	0.999 (0.997 - 1.001)	0.999 (0.998 - 1)
Organ appearance suboptimal * time (days post-transplant)	0.98 (0.96 - 1.00)	**0.998 (0.997 - 0.999)**	0.999 (0.999 - 1)
**Operative characteristics**			
CIT (hours)	**1.04 (1.01 - 1.07)**	**1.03 (1.01 - 1.05)**	**1.02 (1.00 - 1.04)**
Previous abdominal surgery	1.24 (0.97 - 1.58)	**1.20 (1.01 - 1.42)**	1.13 (0.97 - 1.31)
Night-time transplant	1.02 (0.85 - 1.23)	1.01 (0.88 - 1.15)	1.00 (0.89 - 1.12)
Weekend transplant	**0.77 (0.66 - 0.91)**	**0.86 (0.77 - 0.97)**	**0.89 (0.81 - 0.99)**

Table A3 Risk adjusted hazard ratio from Cox regression model for chance of graft failure following liver transplantation in the UK, 1st January 2000 to 31st December 2014

**Risk factor**	**Hazard ratio (95% confidence interval)**		
	**30 days**	**1 year**	**3 years**
**Recipient characteristics**			
Transplant year			
2000	1.00 (-)	1.00 (-)	1.00 (-)
2001	1.17 (0.72 - 1.90)	1.01 (0.70 - 1.46)	0.92 (0.67 - 1.26)
2002	0.95 (0.57 - 1.57)	0.80 (0.54 - 1.17)	0.83 (0.60 - 1.14)
2003	1.17 (0.72 - 1.90)	1.05 (0.72 - 1.51)	0.95 (0.69 - 1.30)
2004	0.97 (0.59 - 1.61)	1.10 (0.77 - 1.57)	0.95 (0.70 - 1.29)
2005	0.88 (0.52 - 1.50)	0.93 (0.64 - 1.37)	0.86 (0.62 - 1.19)
2006	1.15 (0.70 - 1.87)	1.12 (0.78 - 1.59)	0.98 (0.72 - 1.34)
2007	1.00 (0.60 - 1.65)	0.86 (0.59 - 1.26)	0.81 (0.59 - 1.12)
2008	0.78 (0.46 - 1.32)	0.82 (0.57 - 1.20)	0.81 (0.59 - 1.10)
2009	0.81 (0.48 - 1.36)	0.86 (0.59 - 1.25)	0.84 (0.61 - 1.14)
2010	0.79 (0.47 - 1.33)	0.70 (0.48 - 1.03)	**0.64 (0.46 - 0.90)**
2011	0.93 (0.56 - 1.53)	**0.62 (0.42 - 0.92)**	**0.59 (0.42 - 0.83)**
2012	0.64 (0.38 - 1.10)	**0.61 (0.41 - 0.90)**	**0.62 (0.45 - 0.86)**
2013	0.91 (0.56 - 1.48)	0.76 (0.53 - 1.10)	**0.71 (0.52 - 0.98)**
2014	0.73 (0.43 - 1.21)	**0.62 (0.42 - 0.91)**	**0.63 (0.45 - 0.89)**
Age at transplant	**1.01 (1.00 - 1.02)**	**1.01 (1.00 - 1.01)**	**1.00 (1.00 - 1.01)**
Caucasian	0.92 (0.70 - 1.21)	0.87 (0.71 - 1.07)	0.87 (0.73 - 1.03)
Primary liver disease			
Cancer	1.00 (-)	1.00 (-)	1.00 (-)
HCV	0.87 (0.51 - 1.48)	1.12 (0.77 - 1.64)	1.30 (0.95 - 1.77)
Alcohol related liver disease	1.13 (0.69 - 1.85)	1.08 (0.75 - 1.56)	0.97 (0.71 - 1.32)
HBV	1.26 (0.64 - 2.49)	1.18 (0.71 - 1.96)	1.11 (0.72 - 1.71)
PSC	1.51 (0.88 - 2.58)	**1.61 (1.09 - 2.38)**	**1.49 (1.07 - 2.07)**
PBC	1.10 (0.64 - 1.89)	1.09 (0.73 - 1.63)	0.90 (0.64 - 1.27)
Autoimmune hepatitis	1.14 (0.65 - 2.01)	1.09 (0.71 - 1.65)	0.95 (0.66 - 1.36)
Metabolic liver disease	1.58 (0.89 - 2.80)	**1.61 (1.06 - 2.46)**	1.34 (0.93 - 1.93)
Acute liver disease	**1.99 (1.04 - 3.83)**	**2.02 (1.23 - 3.31)**	**1.72 (1.12 - 2.65)**
Re-transplants	**2.26 (1.26 - 4.05)**	**2.48 (1.61 - 3.81)**	**2.29 (1.59 - 3.30)**
Other	1.14 (0.61 - 2.15)	1.15 (0.72 - 1.84)	0.93 (0.62 - 1.40)
Renal support	**1.39 (1.03 - 1.86)**	**1.30 (1.03 - 1.63)**	**1.26 (1.03 - 1.54)**
In-patient	0.99 (0.75 - 1.30)	1.11 (0.91 - 1.34)	1.14 (0.96 - 1.35)

For peer review only

Ventilated	**1.59 (1.08 - 2.35)**	**1.41 (1.04 - 1.92)**	1.28 (0.97 - 1.69)
Oesophageal varices	1.15 (0.94 - 1.40)	1.15 (0.99 - 1.33)	1.07 (0.94 - 1.22)
Presence of TIPS	1.39 (0.90 - 2.15)	**1.45 (1.06 - 2.00)**	1.30 (0.97 - 1.74)
Sepsis confirmed	**1.51 (1.08 - 2.10)**	**1.35 (1.03 - 1.75)**	**1.31 (1.04 - 1.67)**
Acute failure grade prior to transplant	0.84 (0.54 - 1.31)	**0.70 (0.50 - 0.98)**	**0.64 (0.47 - 0.86)**
**Donor characteristics**			
Donor age (years)	1 (0.99 - 1)	**1 (1 - 1.01)**	**1.01 (1.00 - 1.01)**
DCD	**2.22 (1.65 - 2.99)**	**2.18 (1.74 - 2.72)**	**2.07 (1.71 - 2.52)**
Split liver	1.13 (0.65 - 1.96)	**2.33 (1.72 - 3.16)**	**2.32 (1.77 - 3.04)**
Organ appearance suboptimal	**2.74 (2.06 - 3.65)**	**1.85 (1.53 - 2.24)**	**1.73 (1.47 - 2.05)**
Split liver * time (days post-transplant)	**1.05 (1.01 - 1.09)**	0.999 (0.996 – 1.001)	0.999 (0.998 – 1)
Organ appearance suboptimal * time (days post-transplant)	**0.96 (0.94 - 0.99)**	0.998 (0.997 – 1)	0.999 (0.999 - 1)
**Operative characteristics**			
CIT (hours)	**1.04 (1.00 - 1.08)**	**1.03 (1.00 - 1.06)**	**1.02 (1.00 - 1.04)**
Previous abdominal surgery	1.26 (0.95 - 1.67)	**1.23 (1.00 - 1.51)**	1.16 (0.97 - 1.40)
Night-time transplant	1.14 (0.91 - 1.41)	1.07 (0.91 - 1.26)	1.11 (0.97 - 1.28)
Weekend transplant	**0.81 (0.67 - 0.97)**	0.87 (0.76 - 1.01)	0.89 (0.79 - 1.01)

Table A4 Cox regression model for the chance of transplant failure following liver transplantation at night-time compared to daytime

Time from transplant	Unadjusted			Risk adjusted		
	Hazard ratio	95% interval	p-value	Hazard ratio	95% interval	p-value
**30 days**	1.254	1.059 - 1.483	0.009	1.021	0.851 - 1.226	0.8
**One year**	1.247	1.104 - 1.408	0.0004	1.007	0.882 - 1.149	0.9
**Three years**	1.187	1.068 - 1.319	0.002	0.995	0.887 - 1.116	0.9

Table A5 Cox regression model for the chance of graft failure following liver transplantation at night-time compared to daytime

Time from transplant	Unadjusted			Risk adjusted		
	Hazard ratio	95% interval	p-value	Hazard ratio	95% interval	p-value
**30 days**	1.279	1.046 - 1.564	0.02	1.137	0.914 - 1.413	0.2
**One year**	1.227	1.058 - 1.424	0.007	1.071	0.912 - 1.259	0.4
**Three years**	1.233	1.082 - 1.406	0.002	1.112	0.965 - 1.282	0.1

For peer review only

STROBE 2007 (v4) Statement—Checklist of items that should be included in reports of *cohort studies*

Section/Topic	Item #	Recommendation	Reported on page #
**Title and abstract**	1	(*a*) Indicate the study's design with a commonly used term in the title or the abstract	1,2
		(*b*) Provide in the abstract an informative and balanced summary of what was done and what was found	2
**Introduction**			
Background/rationale	2	Explain the scientific background and rationale for the investigation being reported	5
Objectives	3	State specific objectives, including any prespecified hypotheses	6-7
**Methods**			
Study design	4	Present key elements of study design early in the paper	8
Setting	5	Describe the setting, locations, and relevant dates, including periods of recruitment, exposure, follow-up, and data collection	8
Participants	6	(*a*) Give the eligibility criteria, and the sources and methods of selection of participants. Describe methods of follow-up	8
		(*b*) For matched studies, give matching criteria and number of exposed and unexposed	
Variables	7	Clearly define all outcomes, exposures, predictors, potential confounders, and effect modifiers. Give diagnostic criteria, if applicable	9
Data sources/ measurement	8*	For each variable of interest, give sources of data and details of methods of assessment (measurement). Describe comparability of assessment methods if there is more than one group	*8-9*
Bias	9	Describe any efforts to address potential sources of bias	8
Study size	10	Explain how the study size was arrived at10	n/a
Quantitative variables	11	Explain how quantitative variables were handled in the analyses. If applicable, describe which groupings were chosen and why	11-14
Statistical methods	12	(*a*) Describe all statistical methods, including those used to control for confounding	8-9
		(*b*) Describe any methods used to examine subgroups and interactions	7-9
		(*c*) Explain how missing data were addressed	8-9
		(*d*) If applicable, explain how loss to follow-up was addressed	9
		(*e*) Describe any sensitivity analyses	9
**Results**			

For peer review only

Participants	13*	(a) Report numbers of individuals at each stage of study—eg numbers potentially eligible, examined for eligibility, confirmed eligible, included in the study, completing follow-up, and analysed	10
		(b) Give reasons for non-participation at each stage	10
		(c) Consider use of a flow diagram	Na
Descriptive data	14*	(a) Give characteristics of study participants (eg demographic, clinical, social) and information on exposures and potential confounders	10
		(b) Indicate number of participants with missing data for each variable of interest	8, supplementary figure A1
		(c) Summarise follow-up time (eg, average and total amount)	10
Outcome data	15*	Report numbers of outcome events or summary measures over time	10, 11
Main results	16	(*a*) Give unadjusted estimates and, if applicable, confounder-adjusted estimates and their precision (eg, 95% confidence interval). Make clear which confounders were adjusted for and why they were included	11-12
		(*b*) Report category boundaries when continuous variables were categorized	12
		(*c*) If relevant, consider translating estimates of relative risk into absolute risk for a meaningful time period	
Other analyses	17	Report other analyses done—eg analyses of subgroups and interactions, and sensitivity analyses	13-14
**Discussion**			
Key results	18	Summarise key results with reference to study objectives	15
**Limitations**			
Interpretation	20	Give a cautious overall interpretation of results considering objectives, limitations, multiplicity of analyses, results from similar studies, and other relevant evidence	15-17
Generalisability	21	Discuss the generalisability (external validity) of the study results	16-17
**Other information**			
Funding	22	Give the source of funding and the role of the funders for the present study and, if applicable, for the original study on which the present article is based	4

*Give information separately for cases and controls in case-control studies and, if applicable, for exposed and unexposed groups in cohort and cross-sectional studies.

Note: An Explanation and Elaboration article discusses each checklist item and gives methodological background and published examples of transparent reporting. The STROBE checklist is best used in conjunction with this article (freely available on the Web sites of PLoS Medicine at http://www.plosmedicine.org/, Annals of Internal Medicine at http://www.annals.org/, and Epidemiology at http://www.epidem.com/). Information on the STROBE Initiative is available at www.strobe-statement.org.

For peer review only
